# Supplementary material for: Functional hierarchy of the human neocortex across the lifespan
Source: Nature. 2026 Mar 25;652(8111):955–64. doi: 10.1038/s41586-026-10219-x (PMC13102691; doi:10.1038/s41586-026-10219-x)
Supplement: Supplementary file 1 — This file contains Supplementary Results, Discussion and Methods, 15 Supplementary Figures (Supplementary Figs. 1–15) and 3 Supplementary Tables (Supplementary Tables 1–3), with additional analyses, methodological details and supporting visualizations. [file 41586_2026_10219_MOESM1_ESM.pdf]

nature portfolio

https://doi.org/10.1038/s41586-026-10219-x

Supplementary information

Functional hierarchy of the human neocortex across the lifespan

In the format provided by the
authors and unedited

Supplementary Contents

Supplementary Results

1 Gradient Topography and Lifespan Trajectories
2 Global Gradient Metrics
3 RSN Gradient Trajectories
4 Network Interactions
5 Structure-Function Coupling
6 Sex-By-Age Deviations in Global Gradient Metrics
7 Gradients Predict Lifespan Cognition
8 Out-of-Sample Checks in HCP-YA
9 Transcriptomic Enrichment of Gradients

Supplementary Discussion

1 Template Alignment Interpretation
2 Developmental Gradient Ordering
3 Global Gradient Metrics
4 Interpreting Cosine Similarity to the Template
5 Dispersion and FC Degree
6 Resting-State Networks in Gradient Space
7 Transcriptomic Interpretation and Mechanisms

Supplementary Methods

1 Leave-One-Cohort-Out (LOCO) Stability Analysis
2 Bootstrap Validation of WPCA Gradient Template
3 Estimating Trajectory Extrema

Supplementary Figures

Supplementary Tables

Supplementary Results

1 Gradient Topography and Lifespan Trajectories

Sensory–Association (SA) Axis. From mid-adolescence through older age, SA topography is qualitatively stable, with relatively homogeneous values within unimodal regions and progressively stratified values toward transmodal association territories (Figure 2a), consistent with canonical adult SA organization[1]. Correspondingly, the overall SA value distribution exhibits little qualitative change across this interval (Figure 2a).

By contrast, infancy is characterized by attenuated differentiation of the association pole and a more spatially contiguous arrangement of SA extremes (Figure 2a; Extended Data Figure 1). Across childhood, the association pole becomes increasingly focal and stratified, and the loci of association-pole extrema progressively converge toward canonical default-mode territories (Extended Data Figure 2). In parallel, the SA distribution develops heavier tails (Supplementary Figure 6), consistent with increasing separation between vertices occupying unimodal versus transmodal positions along the hierarchy.

Visual–Somatosensory (VS) Axis. Across the lifespan, VS topography is comparatively stable and is dominated by a visual–somatomotor opposition (Figure 2a). Consistent with this relative stability, the most salient developmental changes are expressed as gradual shifts in the strength and asymmetry of pole stratification rather than wholesale reconfiguration of pole identity. At birth, the VS value distribution is approximately symmetric (Extended Data Figure 1); during the first two years, values become denser toward the somatomotor pole, whereas stratification toward the visual pole strengthens through childhood (to $\sim$10 years) (Extended Data Figure 2).

Regionally, the clearest topographic changes involve a gradual migration of superior parietal/dorsal-attention territories toward the visual pole and a complementary drift of auditory and salience/ventral-attention territories toward the somatomotor pole (Figure 2a). After early childhood, VS range and the magnitude of extreme values decrease monotonically, consistent with a progressive reduction in the dominance of modality-specific segregation as large-scale functional organization matures (Figure 2a).

Modulation–Representation (MR) Axis. In adulthood, the MR axis differentiates modulation-oriented cortex (frontoparietal control and attention systems) from representation-oriented regions spanning default-mode and sensory cortices[2,3] (Figure 2a). Relative to SA and VS, MR shows the weakest resemblance to its mature layout at birth, with limited stratification toward either pole (Figure 2a; Extended Data Figure 1). During the first years of life, portions of parietal cortex (overlapping dorsal attention, salience, and control territories) shift toward the modulation pole, but robust pole-specific stratification remains weak at 2 years (Extended Data Figure 2).

Between 2 and 10 years, MR becomes increasingly adult-like as both poles sharpen (Ex-

tended Data Figure 2), indicating a childhood window of differentiation between modulation- and representation-oriented systems. Within the representation pole, visual cortex anchors extreme values during the first year of life, whereas somatosensory cortex approaches the representation pole later, with continued refinement through adolescence and early adulthood (Extended Data Figures 1, 3). This extended timetable is consistent with evidence for protracted development of executive and control-related functions into early adulthood[4,5].

Region-Wise Quantification of Lifespan Effects. To quantify age-related change in gradient value, we fit region-wise GAMMs ($k = 6$ basis functions for the smooth age term) to the mean harmonized gradient value within each of the 400 regions of the Schaefer parcellation[6]. Age effects were widespread across cortex for all axes (SA: $p_{\text{FDR}} < 0.05$ for 99.5% of regions; VS: 100%; MR: 97.25%), with the strongest effects concentrated in somatosensory and default mode parietal cortices for SA, visual and somatosensory cortices for VS, and control-oriented frontal and parietal cortices for MR. Lifespan trajectories were significantly nonlinear for most regions, with the largest fluctuations occurring in regions occupying gradient poles.

2 Global Gradient Metrics

Gradient Range as a Robust Global Measure. To assess the global expression of a gradient, we calculated its range using the inter-vigintile spread (5th–95th percentile) of vertex-wise gradient values. This robustly summarizes the prominence of each axis' connectivity motif while reducing sensitivity to outliers. We fit GAMMs to the SA, VS, and MR ranges and observed significant non-linear, gradient-specific trajectories across the lifespan (Figure 3a). SA and MR undergo gradual expansion throughout infancy, childhood, and adolescence, peaking in early adulthood (SA: 18.8 years, 95% CI 15.0–20.8; MR: 19.0 years, 95% CI 15.5–21.3). During early adulthood, both ranges are relatively stable; in mid and late adulthood SA contracts more rapidly, whereas MR contracts only modestly. Both axes thus exhibit inverted U-shaped development consistent with prior lifespan FC work[7]. In contrast, VS achieves its maximum range during childhood (5.1 years, 95% CI 4.8–5.5) and then gradually contracts across the remainder of the lifespan.

Functional Interpretation of Axis-Specific Range Changes. Because gradient range reflects differentiation in FC profiles between vertices near each pole, expansion of SA indicates increasing dissimilarity between default mode association cortex and primary unimodal cortex (Figure 1a). Similarly, MR expansion reflects increasing contrast between modulation-implicated (frontoparietal control/attention) cortices and representation-implicated (default, sensory, visual, limbic) systems. The VS axis displays divergent global development: its early-childhood peak and subsequent contraction are also evident in the shrinking tails of VS gradient value density distributions and the horizontally contracting embedding plots in the SA–VS plane (Figures 2a,b). This pattern is consistent with reports of decreasing variance explained by VS[8] and a developmental shift from local to long-range FC organization[9]. The protracted expansion of SA and MR aligns with abundant evidence for delayed maturation of heteromodal association cortex and executive/modulatory systems[4,10,11,12].

Gradient Dispersion: Definition and Interpretational Considerations. We quantified how broadly cortical vertices are distributed in the joint 3D embedding by computing gradient dispersion, defined as the average Euclidean embedding distance between each vertex and the embedding centroid for each subject (Figure 3b). Because Euclidean distance in diffusion embedding space approximates geodesic distance in the underlying FC graph, dispersion provides a global measure of FC differentiation with respect to the joint SA–VS–MR embedding: higher values indicate more heterogeneous FC profiles across cortex, whereas lower values indicate reduced cortex-wide diversity in FC profiles. Importantly, dispersion is a scalar summary that pools contributions from all three axes; consequently, it should be interpreted alongside the axis-specific scales (Figure 3a).

We fit a GAMM to mean dispersion versus age (age modeled as a smooth term using splines with $k = 5$ basis functions). Dispersion increases during infancy and childhood, peaks at 13.8 years (95% CI 11.3–16.3), remains relatively stable through early adulthood, and decreases steadily thereafter (Figure 3b). The dispersion peak occurs prior to the SA/MR range peaks in early adulthood, consistent with the fact that VS begins declining in childhood and continues to contract thereafter (Figure 3a). Declining dispersion during later adulthood reflects reduced global differentiation in tandem with contraction of all three axes, consistent with reports of decreasing functional segregation in aging[13,14]. This nonlinear dispersion trajectory provides a normative reference for global differentiation of FC with respect to the first three hierarchical axes of organization across the lifespan.

Template Construction and Cosine Similarity. To compare gradients across subjects, we aligned individual gradient sets to a common lifespan template derived via PCA on gradients from all subjects (SA, VS, MR). To minimize age-related bias in the template, we used a weighted PCA scheme. After alignment, we computed cosine similarity between each subject's aligned gradient and the corresponding template axis, and modeled similarity versus age with GAMMs (Figure 3c). Similarity increased markedly from birth to early adolescence, with maxima achieved first for VS (9.8 years, 95% CI 9.5–12.3), then SA (13.5 years, 95% CI 12.3–14.8), and finally MR (15.8 years, 95% CI 14.8–16.8). This ordering is consistent with the expected maturational timing of sensory/modality-related organization preceding higher-order executive organization and aligns with prior work on the emergence of adult-like network architecture near adolescence[14]. The decline in similarity after mid-adolescence indicates increasing diversity in global FC organization later in life; the sharper VS decline may further suggest reduced sensory-modality dominance in aging.

3 RSN Gradient Trajectories

Rationale and Overview Prior work demonstrates that canonical resting-state networks are stratified along principal FC gradients[1,15] and that developmental change can be described along cortical axes including SA[10,14], with earlier maturation of unimodal systems relative to association, limbic, and attention networks[16,17,18]. To characterize lifespan network reconfiguration in our aligned embedding, we used Schaefer et al.'s 7-network parcellation[6]. For each subject, we summarized each network's position on each gradient by computing the mean aligned gradient

value across network vertices (a network centroid in gradient space). We fit separate GAMMs to each network centroid versus age for each gradient axis.

Global Ordering and Axis-Specific Divergence Across axes, networks were ordered intuitively: default and control networks occupied the association and modulation extremes of SA and MR, respectively, while visual and somatomotor networks occupied the poles of VS. Despite this stable ordering, we observed pronounced axis-dependent and network-dependent trajectories (Figure 4a), indicating that maturation is not a uniform translation of all networks in gradient space but reflects differential timing and directionality relative to each organizing axis.

SA Axis: Hierarchy-Dependent Migration and Early Somatomotor Deviation Changes in SA network configuration are naturally interpreted in the context of SA range expansion during development and contraction during aging (Figure 3a). Movement toward the association pole can be interpreted as increased embedding of a network within transmodal, default-associated connectivity motifs, whereas movement toward the sensory pole reflects greater integration with the unimodal base of the sensory–association hierarchy. In this framing, default and control networks migrate toward the association pole from birth to mid-adolescence (Figure 4a, left), consistent with late-emerging transmodal architecture. In contrast, salience/ventral attention, dorsal attention, and visual networks move down the SA axis more gradually through early adulthood and become more central thereafter. The somatomotor network exhibits a distinct early-life trajectory (Figure 4c): it begins at the sensory extreme at birth, moves centrally by $\sim$3 years, and then converges with attention/visual trajectories. This pattern is plausibly related to infancy-associated SA topographic reorganization, in which the sensory pole is initially concentrated in somatomotor cortex and later becomes more balanced with the visual system (Figure 2a).

VS Axis: Early Maximal Separation and Later Contraction The VS axis distinguishes preferential involvement in visual versus somatosensory processing streams. Accordingly, heteromodal networks (default, control, limbic, dorsal attention) occupy central VS positions across the lifespan (Figure 4a, middle), while visual and somatomotor networks occupy opposite poles and salience/ventral attention lies on the somatomotor side, suggesting a strong somatomotor component in this network's functional profile. Network separation is greatest in infancy and early childhood (Figure 4a, middle): the somatomotor centroid is most extreme at birth and trends centrally across the lifespan, whereas the visual network is already strongly differentiated along this axis at birth and remains near the visual pole throughout development. Salience/ventral attention moves toward the somatomotor pole from birth to $\sim$10 years, increasing its preferential involvement in somatomotor-related processing during this period. The pole networks (visual, somatomotor, salience/ventral attention) are maximally separated between birth and $\sim$10 years and contract rapidly thereafter, paralleling global VS range contraction (Figure 3a).

MR Axis: Heterogeneous and Protracted Network-Specific Timing MR dynamics are the most heterogeneous (Figure 4a, right), consistent with MR differentiating modulation-implicated versus
representation-implicated systems. In this organization, control and attention networks reside toward the modulation pole, while default and unimodal networks lie toward representation.

Default and control separate progressively from birth through early adulthood, driven by rapid early migration of default toward representation (birth to $\sim$5 years) and a sustained migration of control toward modulation through early adulthood. In adulthood, the control network shifts centrally, coincident with MR contraction (Figure 2b). Attention networks show dissociable timing: dorsal attention reaches maximal modulation-pole positioning in early adolescence, whereas salience/ventral attention exhibits more protracted development, reaching its maximum in mid adulthood.

4 Network Interactions

To examine how canonical resting-state networks reorganize relative to one another in gradient space, we quantified between-network dispersion as the mean Euclidean distance between all vertex-to-vertex pairs belonging to two Schaefer-7 networks in each subject's aligned embedding (Extended Data Figure 4). This metric captures both translational shifts and shape changes of the network clouds and therefore offers a direct read-out of functional segregation (larger values) or integration (smaller values) between systems.

Across infancy and early childhood most network pairs diverge sharply. Visual connectivity is the earliest to pull away: dispersion between the visual network and control, default, salience/ventral-attention, and limbic systems rises steeply from birth and reaches a plateau by roughly ten years, reflecting early functional differentiation. By contrast, dispersion that involves somatomotor nodes follows a slower timetable. Somatomotor links to transmodal partners (somatomotor–default, somatomotor–control) stay essentially flat during the first five years, surge between childhood and late adolescence, and only then decline, suggesting a delayed but pronounced period of segregation for sensorimotor circuits.

High-order interactions among default, control, and salience/ventral-attention networks continue to separate through adolescence, reaching maximal distances near the mid-twenties before gradually reconverging. Visual–somatomotor dispersion shows a complementary profile: a rapid childhood increase is followed by a steady contraction from adolescence into late adulthood, indicating that early differentiation along the VS axis eventually yields to reintegration.

Taken together, these trajectories underscore that network reorganization is coupled tobut not identical withthe global gradient dynamics described above. Visual segregation emerges first, transmodal refinement continues longest, and somatomotor segregation both appears later and recedes earlier than most other interactions. In adulthood all network pairs decline in dispersion, with the most striking late-life contraction occurring between visual and somatomotor systems. For completeness we repeated the analysis using the centroid-to-centroid distance originally proposed by Bethlehem et al.[19], and found that the resulting curves preserve the overall ordering of network pairs (Supplementary Figure 9).

5 Structure-Function Coupling

Structural Features and Gradient Estimation Structural gradients were computed from affinity matrices based on pairwise Pearson correlation of cortical features, including cortical thickness, myelination, tissue volume fractions (intra-cellular, extra-cellular, intra-soma), and diffusion-derived metrics (fractional anisotropy, mean diffusivity, microscopic fractional anisotropy, microscopic mean diffusivity, microscopic anisotropy index, and orientation coherence index). Structural gradients were computed for each subject using the same embedding procedure as functional gradients, yielding a set of axes of microstructural organization for each individual. Each subject's structural embedding was then aligned to their corresponding aligned functional gradients (SA, VS, MR), enabling axis-matched comparisons.

Topographic Differences After Alignment Even after Procrustes alignment, structural gradients were not topographically identical to functional gradients (Figure 5a). Qualitatively, the SA structural and functional gradients exhibited the clearest correspondence. In contrast, the VS-aligned structural gradient differed substantially from the functional VS axis: while visual cortex occupied the visual extreme in both embeddings, somatosensory cortex did not form the opposing pole of the VS-aligned structural gradient. This indicates that microstructural contrasts between visual and somatosensory systems do not coincide with the functional VS axis. The MR-aligned structural gradient also diverged from its functional counterpart, showing reduced localization at the modulation pole and lacking the DMN-associated representation pole observed in the functional MR axis. Collectively, these observations support the interpretation that microstructural organization most strongly underpins SA, with weaker orthogonal microstructural gradients corresponding to VS and MR.

Coupling Trajectories and Structural Gradient Scale Axis-specific structure–function coupling was quantified as cosine similarity between aligned structural and functional gradients (Figure 5b). Coupling decreased nonlinearly with age (Figure 5c). Declines were steepest for SA and MR during infancy and early childhood, whereas VS coupling was relatively stable through infancy and childhood and declined more modestly after $\sim$10 years. Structural gradient ranges exhibited lifespan trajectories that broadly mirrored functional axes but with distinct timing (Figure 5d). Specifically, the SA-aligned structural gradient range peaked later at 39.3 years (95% CI 14.8–63.8 years), the VS-aligned structural gradient range peaked early at 3.4 years (95% CI 2.3–4.5 years), and the MR-aligned structural gradient range peaked at 13.7 years (95% CI 9.5–17.8 years). Structural gradient dispersion peaked earlier than functional gradient dispersion, reaching 7.3 years (95% CI 7.0–7.6 years), consistent with earlier maximal differentiation in the joint structural embedding. Network-level summaries of structure–function coupling and aligned structural gradient values (Schaefer-7 networks) are shown in Supplementary Figure 10.

Metric-Specific Coupling Along the SA Hierarchy We also examined how the SA axis relates to individual microstructural metrics across development (Extended Data Figure 5). Negative correlations indicate higher metric values at the sensory pole, while positive correlations indicate higher values at the association pole. Metrics reflecting axonal/myelin development showed strong negative coupling: myelination (T1w/T2w) exhibited the most persistent alignment with

SA, peaking in early adulthood before modest decline[20,21], and intra-cellular volume fraction also showed strong negative coupling peaking earlier in childhood. In contrast, cortical morphology and extra-cellular space metrics were mainly positively coupled to SA: cortical thickness exhibited robust positive coupling peaking in adolescence, consistent with thicker association cortex[22], and extra-cellular volume fraction followed a similar positive trajectory after a transient neonatal negative correlation. Several metrics showed developmental sign changes, including FA (positive to negative), intra-soma volume fraction (negative to positive), and free-water fraction (strong positive to weak negative), indicating that the microstructural correlates of the SA hierarchy vary across developmental epochs. These axis-locked patterns support the view that structural development proceeds along SA and is related to functional hierarchy formation, while the coupling between structure and function evolves across the lifespan[23,24].

6 Sex-By-Age Deviations in Global Gradient Metrics

We assessed sex-related effects on global gradient metrics using a two-step GAMM procedure. After fitting a sex-agnostic lifespan trajectory for each metric and computing residuals, we modeled these residuals with sex-specific smooth functions of square-root age to evaluate whether male and female trajectories exhibit systematic, age-dependent departures from the population mean trajectory (Supplementary Fig. 1a,c). Using the p-values of the sex-specific smooth terms, we observed significant age-dependent deviations for SA range and for gradient dispersion, whereas VS range and MR range showed little to no evidence for systematic sex-dependent deviations. Although these deviations were statistically significant for dispersion and SA range, the reconstructed male and female trajectories remained qualitatively similar, suggesting that sex effects are modest in magnitude. Overall, sex-related effects on global gradient metrics were limited in scope and were most evident for SA range and global dispersion. Additionally, we computed spatial maps comparing lifespan-mean gradients in males and females (Supplementary Fig. 1b). Females showed higher values at the association pole and lower values at the somatosensory pole of the SA axis, consistent with greater differentiation along the SA axis in females.

7 Gradients Predict Lifespan Cognition

Effect Definition for Extended Data Figure 6a. For each gradient–domain pair, the main effect at the pooled median age (30 years) was quantified as the predicted cognitive difference between individuals at the 90th and 10th percentiles of the gradient metric, holding covariates constant. This yields an interpretable high–low contrast that can be compared across metrics and domains (Extended Data Figure 6a).

Age Modulation for Extended Data Figure 6b. Panel **b** encodes how the high–low gradient contrast changes from the 10th to the 90th age percentile (positive values indicate strengthening with age; negative values indicate weakening). This representation isolates the age-dependent component of each gradient–cognition association and highlights consistent lifespan trends across domains (Extended Data Figure 6b).

Cohort-Specific Models and Tested Metrics. In both cohorts, we tested the same family of FC-gradient metrics: global dispersion; axis-specific ranges; cosine similarity to the canonical SA, VS, and MR axes; and the first three gradient eigenvalues, which are alignment-agnostic. In BCP, we fit age-adjusted random-intercept linear mixed-effects models for each Mullen domain score and each gradient metric, controlling for participant as a random effect (Supplementary Table 1; Extended Data Figure 6d). Across 60 tests, four associations survived FDR correction ($p_{\text{FDR}} < 0.05$): MR cosine similarity positively predicted Visual Reception, Receptive Language, and Composite scores ($\beta = 0.04$–0.05; $p_{\text{FDR}} = 0.021$), and the third eigenvalue predicted Fine Motor ability ($\beta = 0.05$; $p_{\text{FDR}} = 0.021$).

In HCP–YA, linear models identified 35 of 90 gradient–cognition associations surviving FDR correction (Supplementary Table 2; Extended Data Figure 6c). The most consistent predictor was SA cosine similarity, which showed significant positive associations across all cognitive domains. Dispersion and SA range were also strongly associated with composite measures of total and fluid cognition (Supplementary Table 2).

8 Out-of-Sample Checks in HCP-YA

Out-of-sample checks within HCP–YA were directionally consistent: adding SA fidelity as an age interaction term yielded a median $\Delta R^2 = 0.017$ across the nine NIH Toolbox outcomes (IQR: 0.008 – 0.025), with total composite $\Delta R^2 = 0.028$ (IQR: -0.035 – 0.090). These gains are modest, as expected for resting-state predictors in healthy young adults, but reproducible across domains.

9 Transcriptomic Enrichment of Gradients

PLS Framework and Orientation At each selected age (0.5, 2, 10, 25, 40, 80 years), we related parcel-wise mean gradient values to adult cortical gene expression (Allen Human Brain Atlas[25] processed with abagen[26] on the Schaefer-400 parcellation) using a one-component PLS model. For interpretability across ages, we oriented the first PLS component such that the correlation between its parcel score and the mean gradient value was non-negative at each age. Regions with higher PLS scores thus correspond to expression profiles more aligned with higher gradient values, whereas low-scoring regions align with lower gradient values.

Gene Ranking, GO Enrichment, and Gene-Set Projection For each axis and age, we ranked genes by their PLS coefficients and performed GO enrichment using GOrilla[27]. To visualize how specific enriched terms map onto cortex, we computed parcel-wise gene-set scores for selected GO terms as coefficient-weighted means of z-scored expression across member genes, and compared these maps to the corresponding PLS component maps (Extended Data Figures 8b, 9b, 10b). To summarize many GO terms, we grouped enriched terms into coarse biological themes using keyword rules and tracked theme enrichment across ages (Extended Data Figures 8c, 9c, 10c).

Significance Testing of Gene–Gradient Coupling For each axis and age, we quantified gene–gradient coupling as the correlation between PLS parcel scores and the corresponding mean gradient values. Significance was assessed by permutation testing and corrected across ages using Benjamini–Hochberg, yielding lifespan coupling trajectories for SA, VS, and MR (Extended Data Figures 8d, 9d, 10d).

Axis-Specific Interpretations For SA, synaptic signaling and vesicle-cycle programs were consistently enriched across ages, while additional themes (e.g., ion transport/excitability, extracellular matrix/adhesion/migration, immune/microglia, and cell cycle/proliferation) appeared more selectively at particular developmental stages (Extended Data Figure 8c). For VS, enriched terms emphasized RNA metabolism/transcription and neurite/transport processes, with vesicle-cycle enrichment strongest early and transcription/RNA-metabolism enrichment peaking in young to mid-adulthood (Extended Data Figure 9c). For MR, ion transport/excitability was the most consistent theme and showed strongest enrichment in late life (Extended Data Figure 10c), suggesting a selective contribution of homeostatic/excitability programs to the control-dominated pole of the MR molecular axis.

Supplementary Discussion

1 Template Alignment Interpretation

The use of a template gradient set and subsequent alignment of individual subject gradients to that template was crucial to standardizing gradient architecture across the lifespan. However, this strategy presupposes that some global organizational features of connectivity are sufficiently conserved to support a shared coordinate system across age. The computation of a global lifespan template is inherently biased toward periods of stable FC gradients. As a result, alignment of infant and childhood gradient sets to a lifespan template yields the best representation of the adult connectivity features present in each infant or child, irrespective of whether those features are the principal organizing motifs in early life. In this sense, our analysis emphasizes how FC in early life converges toward an adult-like architecture, rather than fully characterizing the native organizing principles of the infant brain themselves. Importantly, this does not force early-life gradients to look adult-like; rather, it prioritizes extracting the adult-referenced component of early-life organization (which can still be weak, rotated, or spatially redistributed). This point is underscored by the marked late-life decline in axis-wise cosine similarity (especially for VS; Figure 3c), showing that alignment does not enforce canonical topography when subject-level gradient structure deviates from the template. Although subtle, this distinction is important for interpretation and motivates complementary analyses (e.g., age-specific templates/embeddings, or principal-angle/procrustes measures of low-dimensional subspace stability) that do not presuppose one-to-one axis correspondence.

2 Developmental Gradient Ordering

Our observation that the global range of the VS axis decreases throughout late childhood and adolescence is consistent with previous work[28,29]. However, our results differ in that we do not observe robust reordering of the dominant gradient axes during development, in contrast to studies reporting axis reordering during early adolescence[28]. Notably, changes in component rank can reflect rotation within a relatively stable low-dimensional subspace, particularly when leading components explain similar variance, rather than a qualitative change in the underlying embedding geometry. This discrepancy may therefore reflect methodological differences: our framework uses group alignment across the entire lifespan followed by GAMM fitting to characterize continuous topographic and global changes in each axis, whereas prior work relied on age-binned analyses. Under age-binning approaches, ordering can be sensitive to cohort composition, preprocessing differences, and the extent to which axes are allowed to rotate or swap in sign and rank across bins. In contrast, lifespan-wide alignment encourages continuity of the canonical axes and facilitates direct comparison of axis-specific trajectories, at the potential cost of under-representing transient or age-specific motifs. Future work that explicitly compares age-binned and lifespan-aligned pipelines within the same datasets, and that evaluates subspace stability independent of axis labeling, may help clarify when apparent reordering reflects genuine developmental change versus methodological instability.

3 Global Gradient Metrics

Our analysis of global gradient metrics across the lifespan yielded several results that help interpret the development of global hierarchical FC architecture. Here, gradient range is axis-specific, whereas dispersion summarizes the joint spread of vertices in low-dimensional embedding space independent of any particular axis. A monotonic increase in dispersion between birth and 13.8 years (Figure 3b; the dispersion peak in our sample) indicates that global connectivity differentiation with respect to the SA, VS, and MR axes expands during this period. Declining gradient range of the VS axis during the same period (Figure 3a), however, underscores that increasing dispersion is driven primarily by expanding transmodal SA and MR axes. It is also notable that increases in gradient dispersion are modest between birth and 13.8 years, a developmental stage well-known for rapid differentiation of cortical FC[30,31]. In tandem, expanding gradient range during this period in combination with relatively stable dispersion indicates that the extreme values along each gradient increase while not driving a commensurate change in the average spread of vertices in embedding space.

One potential interpretation is that increased prominence of transmodal hubs at the association and modulation poles of the SA and MR axes occurs alongside increasing integration among intermediary and specialized cortical locations. Migration of hubs toward more extreme positions in embedding space engenders increasing gradient range, while increased integration among intermediary nodes constrains the mean global spread (dispersion). We also observe monotonic decline in global gradient dispersion after 13.8 years, indicating decreasing FC differentiation during aging. This is consistent with observations that functional segregation decreases during later adulthood, while within-system FC and between-system FC decrease and increase, respectively[13,32].

4 Interpreting Cosine Similarity to the Template

Cosine similarity between each subject's aligned gradient map and the template provides an axis-specific, scale-invariant index of topographic fidelity. Because cosine similarity is insensitive to overall gradient magnitude, it is not explained by axis contraction alone (e.g., decreasing range). Instead, reductions in cosine similarity indicate that the spatial pattern of the corresponding axis is less canonical, either because the axis is genuinely reconfigured or because it is estimated with reduced stability.

Across the lifespan, cosine similarity exhibits a pronounced decline in later adulthood, most prominently for the VS axis (Figure 3c). This pattern suggests that the canonical visual–somatosensory differentiation becomes increasingly heterogeneous across individuals with age. A key geometric consideration is that alignment is performed jointly across the low-dimensional embedding: when one axis becomes less distinctive, the optimal alignment can be dominated by the remaining axes, leaving the weakest axis with greater residual mismatch. Practically, these findings indicate that late-life VS organization is captured reliably at the population level but is less uniformly instantiated at the individual level, as reflected by the increasing variance and

declining mean similarity.

5 Dispersion and FC Degree

Many previous studies have studied FC organization via graph-theoretic metrics[33,34,35], while comparatively little effort has been made to establish connections between FC gradients and underlying graph architecture. We sought to partially address this gap by studying relationships between metrics derived from diffusion embedding and conventional summaries of whole-brain FC organization. In particular, we examined the relationship between gradient dispersion—a measure of global connectivity differentiation in the joint embedding independent of any particular axis—and mean FC degree (mean nodal strength), a global measure of overall FC magnitude. We observed a moderate positive association between dispersion and mean FC degree across subjects (Figure 3d), indicating that participants with more differentiated embeddings also tend to exhibit higher overall FC strength.

From a graph/embedding perspective, mean degree indexes the overall weight of the functional affinity graph, while dispersion indexes how heterogeneously that weight is distributed across nodes. A positive association between the two implies that subjects with globally stronger FC also show more pronounced structure in the similarity graph, yielding embeddings with larger global spread. Because mean FC strength can be sensitive to preprocessing choices and global confounds (e.g., denoising and motion), this association should be interpreted descriptively and its robustness to alternative pipelines represents an important direction for future work.

6 Resting-State Networks in Gradient Space

We also characterized how gradient organization evolves across the human lifespan with respect to canonical resting-state networks (RSNs). Using the 7-network parcellation of Schaefer et al.[6], we examined the trajectories and distributions of network vertices in gradient space, revealing a complex sequencing of network development with respect to SA, VS, and MR. Network trajectories along the SA and VS axes are comparatively simple: motion of each network along these axes coincides with changes in SA and VS range, driving expansion and contraction of separation between networks at either pole, with little evidence for large-scale reordering. Beyond centroid motion, age-related changes in within-network spread and between-network overlap in gradient space provide a natural geometric interpretation of dedifferentiation.

In contrast, network centroids along the MR axis exhibited substantial reordering with differential timing across networks, consistent with the MR axis demanding greater topographic reorganization across development and maturing later than SA and VS. Notably, dorsal and ventral attention networks (DAN and VAN) differed in their developmental timing on the MR axis, with DAN reaching its maximal MR value earlier than VAN. One interpretation is that refinement of top-down attention mechanisms associated with DAN may be most prominent earlier in

development, with bottom-up attention systems linked to VAN unfolding more gradually. Further work examining the differential development and interaction of these attention systems may clarify how MR-axis maturation supports the emergence and maintenance of cognitive control across the lifespan.

7 Transcriptomic Interpretation and Mechanisms

To anchor developmental patterns in underlying biology, we related parcel-wise adult cortical expression (AHBA; abagen) to the mean gradient at each age using supervised PLS. Because the Allen Human Brain Atlas is derived from adult post-mortem donors, these PLS components should be interpreted as adult-like molecular axes that we evaluate against age-varying functional gradients, rather than as direct readouts of developmental changes in gene expression. Across all three axes, gene–gradient coupling was generally strongest early in life and weaker in later adulthood. This pattern is consistent with a developmental regime in which adult-like spatiomolecular gradients exert strong constraints on early functional topography, which is then progressively refined by experience-dependent plasticity, structural maturation, and age-related change. As with any covariance-based approach, PLS identifies spatial correspondence rather than mechanism.

The SA, VS, and MR axes revealed partially dissociable molecular programs. For the SA axis, the molecular axis closely followed the classic sensory–association hierarchy and was consistently enriched for synaptic signaling and vesicle-related processes, supporting the idea that SA organization is rooted in regional differences in synaptic machinery and vesicle trafficking. Along the VS axis, the molecular component peaked in visual cortex and implicated transcriptional/RNA-metabolic and transport-related programs with a visual emphasis, consistent with molecular specialization associated with visual–somatosensory differentiation. In contrast, the MR axis was associated with a molecular gradient loading negatively in visual and premotor cortex and positively in medial and lateral prefrontal and anterior temporal regions, with dominant signals involving ion transport/excitability and metal-ion handling. Notably, enrichment of ion transport and homeostatic themes along MR resonates with work linking regional intrinsic timescales to ion-channel and excitation–inhibition-related genes[36]. This convergence raises the hypothesis that MR partly reflects a dimension of temporal integration capacity supporting sustained modulatory computations in transmodal cortex. Future work could test this account by examining whether intrinsic timescale maps and independently derived spatiomolecular gradients preferentially align with MR- and SA-related molecular axes.

Our findings also fit within, and extend, recent work on whole-brain spatiomolecular gradients. Vogel et al.[37] and Xia et al.[29] argued that spatiomolecular axes constitute a developmental scaffold for adult functional specialization. Here, by focusing on three well-characterized functional gradients and tracking their coupling to transcriptomic axes across the lifespan, we suggest that this scaffold is not uniform but differentiated into at least three partially dissociable molecular programs corresponding to SA, VS, and MR. Together with the early-life peak in gene–gradient coupling, these results support a view in which molecular constraints are strongest during early differentiation and become progressively less predictive as functional networks mature

and diversify.

Supplementary Methods

1 Leave-One-Cohort-Out (LOCO) Stability Analysis

Four cohorts in our analysis (HCPD and three site-specific subsets of HBN, denoted HBNA/B/C) overlap in age with at least one other cohort. We assessed the extent to which any single cohort could influence the vertex-wise lifespan trajectories of functional connectivity (FC) gradient values. To do this, we repeated the full harmonization and GAMM pipeline (see Methods: Lifespan Trajectories) four times, each time withholding one cohort and refitting the model at every vertex. All preprocessing, weighting, and modeling parameters were held constant, matching the primary analysis, to ensure that any differences observed could be attributed solely to the exclusion of that cohort. From the resulting four fitted trajectories, we computed the following metrics for each vertex:

1. The standard deviation across folds, averaged over the trajectory;
2. The peak-to-trough amplitude of the mean LOCO curve;
3. The absolute change in amplitude compared to the full-data fit, reported in both raw units and as a percentage of the full-data amplitude; and
4. The absolute change in the age at which the vertex reached its maximum value.

Supplementary Figure 11 presents example LOCO curves for selected vertices, with the harmonized gradient values overlaid for reference. To assess out-of-sample (OOS) generalizability, we harmonized the withheld cohort to the non-withheld cohorts by matching mean and variance. For each vertex, we computed the root-mean-square error (RMSE) and OOS R^2 between predicted and harmonized values, and the Spearman correlation between residuals and age.

Across the SA axis (18,644 vertices), the median standard deviation of the LOCO trajectories was 0.056 gradient units (IQR = 0.040–0.078; 95th percentile = 0.125), compared to a median peak-to-trough amplitude of 0.821 unitsindicating that typical LOCO variability was 6.8% of the signal amplitude. The VS and MR axes showed similar or lower sensitivity (VS: median SD = 0.032, amplitude = 0.665, relative SD = 4.8%; MR: median SD = 0.029, amplitude = 0.435, relative SD = 6.6%). Median changes in amplitude relative to the full-data fit were 6.1% (SA), 6.0% (VS), and 4.6% (MR). For 75% of vertices, the age of peak gradient expression shifted by no more than 0.75 years. OOS residuals were effectively uncorrelated with age (median $\rho \approx 0$, IQR $\pm$ 0.07), and RMSE distributions were tightly centered (SA: 0.775; VS: 0.496; MR: 0.462). These results indicate that no single cohort disproportionately influenced the developmental trajectories, and that the harmonized GAMM fits generalize well to held-out data.

2 Bootstrap Validation of WPCA Gradient Template

To verify that inverse-frequency weighting corrects for the sampling bias introduced by our uneven age distribution, we compared the canonical WPCA template used in our main analysis (the Procrustes alignment target) with templates derived from strictly age-balanced subsamples. Chronological age was first transformed with an exponent of $\alpha = 0.50$, and then divided into ten equal-width bins in the transformed space. On the raw age scale, this expands early-life intervals and compresses later-life intervals, thereby emphasizing periods of rapid neurodevelopment. For each of $B = 500$ bootstrap replicates, we randomly sampled 50 participants per bin, performed an unweighted principal component analysis (PCA) on the subject-by-gradient matrix, and greedily aligned the first three principal components to the WPCA template by maximizing absolute spatial correlation and enforcing consistent sign.

Template similarity was assessed in two complementary ways. First, we computed the three-dimensional subspace angle (θ, in degrees) between each bootstrap-derived template and the WPCA template using the average of the principal angles obtained based on Procrustes alignment. Second, we calculated the Pearson correlation between each matched axis and its WPCA counterpart. Since the canonical template was generated using inverse-frequency weighting, small θ values and near-unity axis correlations would indicate successful correction of age-related sampling bias. Supplementary Figure 12 shows the distributions of subspace angles and axis correlations between the balanced and canonical templates.

Across the 500 balanced datasets, the mean subspace angle was $6.18° \pm 0.52°$ (median = 6.15°, IQR = 5.82–6.50°, 95% interval = 5.30–7.27°). Axis correlations were consistently high, confirming preservation of major variance directions: PC1 = 0.995 ± 0.001 (median = 0.995, IQR = 0.995–0.996), PC2 = 0.955 ± 0.012 (median = 0.956, IQR = 0.948–0.963), and PC3 = 0.944 ± 0.013 (median = 0.945, IQR = 0.936–0.954). These tightly bounded estimates demonstrate that the WPCA template derived from the full, unevenly sampled cohort is statistically indistinguishable from one derived using age-balanced subsamples. Inverse-frequency weighting thus offers an effective and computationally efficient alternative to explicit resampling when estimating population-level principal components in lifespan neuroimaging studies.

3 Estimating Trajectory Extrema

In several cases, it was of interest to obtain estimates of the age at which gradient-derived metrics reached maxima or minima in their lifespan trajectories as an estimate of their maturation age. To obtain uncertainty estimations for the ages at which each metric reached its maximum or minimum value, we used a bootstrapping procedure. We drew 20,000 samples from the posterior distribution of the smooth term's (age) coefficients, reflecting uncertainty in the model estimates. Each sample represented a possible realization of the age-metric relationship, from which we calculated the fitted metric values across the age sequence. For each bootstrap sample, we identified the age at which the metric achieved its maximum (or minimum) value, thus creating a distribution of ages corresponding to these extreme values across all samples. The 95% confidence

intervals (CIs) were then computed from this distribution, providing a probabilistic range within which the true age of the maximum (or minimum) was most likely to be found.

Supplementary References

1. Margulies, D. S. *et al.* Situating the default-mode network along a principal gradient of macroscale cortical organization. *Proceedings of the National Academy of Sciences of the United States of America* **113,** 12574–12579 (2016).
2. Power, J. D. & Petersen, S. E. Control-related systems in the human brain. *Current Opinion in Neurobiology* (2013).
3. Zhang, J. *et al.* Intrinsic functional connectivity is organized as three interdependent gradients. *Scientific Reports* (2019).
4. Parlatini, V. *et al.* Functional segregation and integration within fronto-parietal networks. *NeuroImage* (2017).
5. Pozuelos, J. P., Paz-Alonso, P. M., Castillo, A., Fuentes, L. J. & Rueda, M. R. Development of attention networks and their interactions in childhood. *Developmental Psychology* **50,** 2405–2415 (2014).
6. Schaefer, A. *et al.* Local-global parcellation of the human cerebral cortex from intrinsic functional connectivity MRI. *Cerebral Cortex* (2018).
7. Abrol, A. *et al.* Developmental and aging resting functional magnetic resonance imaging brain state adaptations in adolescents and adults: A large N (>47K) study. *Human Brain Mapping* **44,** 2158–2175 (2023).
8. Larivière, S. *et al.* Multiscale structure-function gradients in the neonatal connectome. *Cerebral Cortex* **30,** 47–58 (2020).
9. Fair, D. A. *et al.* Functional brain networks develop from a "local to distributed" organization. *PLoS Computational Biology* (2009).
10. Sydnor, V. J. *et al.* Intrinsic activity develops along a sensorimotor-association cortical axis in youth. *Nature Neuroscience* **26,** 638–649 (2022).
11. Fair, D. A. *et al.* Development of distinct control networks through segregation and integration. *Proceedings of the National Academy of Sciences of the United States of America* (2007).
12. Vogel, A. C., Power, J. D., Petersen, S. E. & Schlaggar, B. L. Development of the brain's functional network architecture. *Neuropsychology Review* **20,** 362–375 (2010).
13. Chan, M. Y., Park, D. C., Savalia, N. K., Petersen, S. E. & Wig, G. S. Decreased segregation of brain systems across the healthy adult lifespan. *Proceedings of the National Academy of Sciences of the United States of America* (2014).
14. Sun, L. *et al.* Human lifespan changes in the brain's functional connectome. *Nature Neuroscience* **28,** 891–901 (2025).
15. Katsumi, Y. *et al.* Correspondence of functional connectivity gradients across human isocortex, cerebellum, and hippocampus. *Communications Biology* **6,** 1–13 (2023).
16. Supekar, K., Musen, M. & Menon, V. Development of large-scale functional brain networks in children. *PLoS Biology* (2009).
17. Casey, B. J., Tottenham, N., Liston, C. & Durston, S. Imaging the developing brain: What have we learned about cognitive development? *Trends in Cognitive Sciences* (2005).

18. Menon, V. Developmental pathways to functional brain networks: Emerging principles. *Trends in Cognitive Sciences* (2013).
19. Bethlehem, R. A. *et al.* Dispersion of functional gradients across the adult lifespan. *NeuroImage* **222,** 117299 (2020).
20. Glasser, M. F. *et al.* A multi-modal parcellation of human cerebral cortex. *Nature* (2016).
21. Glasser, M. F. & Van Essen, D. C. Mapping human cortical areas in vivo based on myelin content as revealed by T1-and T2-weighted MRI. *Journal of Neuroscience* **31,** 11597–11616 (2011).
22. Frangou, S. *et al.* Cortical thickness across the lifespan: Data from 17,075 healthy individuals aged 3–90 years. *Human Brain Mapping* **43,** 431–451 (2022).
23. Baum, G. L. *et al.* Development of structure-function coupling in human brain networks during youth. *Proceedings of the National Academy of Sciences of the United States of America* **117,** 771–778 (2020).
24. Dubois, J. *et al.* The early development of brain white matter: a review of imaging studies in fetuses, newborns and infants. *Neuroscience* **276,** 48–71 (2014).
25. Hawrylycz, M. J., Lein, E. S., Guillozet-Bongaarts, A. L., *et al.* An anatomically comprehensive atlas of the adult human brain transcriptome. *Nature* **489,** 391–399 (2012).
26. Markello, R. D. *et al.* Standardizing workflows in imaging transcriptomics with the abagen toolbox. *eLife* **10,** e72129 (2021).
27. Eden, E., Navon, R., Steinfeld, I., Lipson, D. & Yakhini, Z. GOrilla: a tool for discovery and visualization of enriched GO terms in ranked gene lists. *BMC Bioinformatics* **10,** 48 (2009).
28. Dong, H. M., Margulies, D. S., Zuo, X. N. & Holmes, A. J. Shifting gradients of macroscale cortical organization mark the transition from childhood to adolescence. *Proceedings of the National Academy of Sciences of the United States of America* (2021).
29. Xia, Y. *et al.* Development of functional connectome gradients during childhood and adolescence. *Science Bulletin* **67,** 1049–1061 (2022).
30. Wen, X. *et al.* First-year development of modules and hubs in infant brain functional networks. *NeuroImage* (2019).
31. Gao, W., Alcauter, S., Smith, J. K., Gilmore, J. H. & Lin, W. Development of human brain cortical network architecture during infancy. *Brain Structure and Function* (2015).
32. Betzel, R. F. *et al.* Changes in structural and functional connectivity among resting-state networks across the human lifespan. *NeuroImage* **102,** 345–357 (2014).
33. Van den Heuvel, M. P. & Sporns, O. Network hubs in the human brain. *Trends in Cognitive Sciences* **17,** 683–696 (2013).
34. Sporns, O. & Betzel, R. F. Modular brain networks. *Annual Review of Psychology* (2016).
35. Bassett, D. S. & Bullmore, E. T. Small-world brain networks revisited. *Neuroscientist* **23,** 499–516 (2017).
36. Gao, R., van den Brink, R. L., Pfeffer, T. & Voytek, B. Neuronal timescales are functionally dynamic and shaped by cortical microarchitecture. *eLife* **9,** e61277 (2020).

37. Vogel, J. W. *et al.* Deciphering the functional specialization of whole-brain spatiomolecular gradients in the adult brain. *Proceedings of the National Academy of Sciences of the United States of America* **121,** e2219137121 (2024).

Supplementary Figures

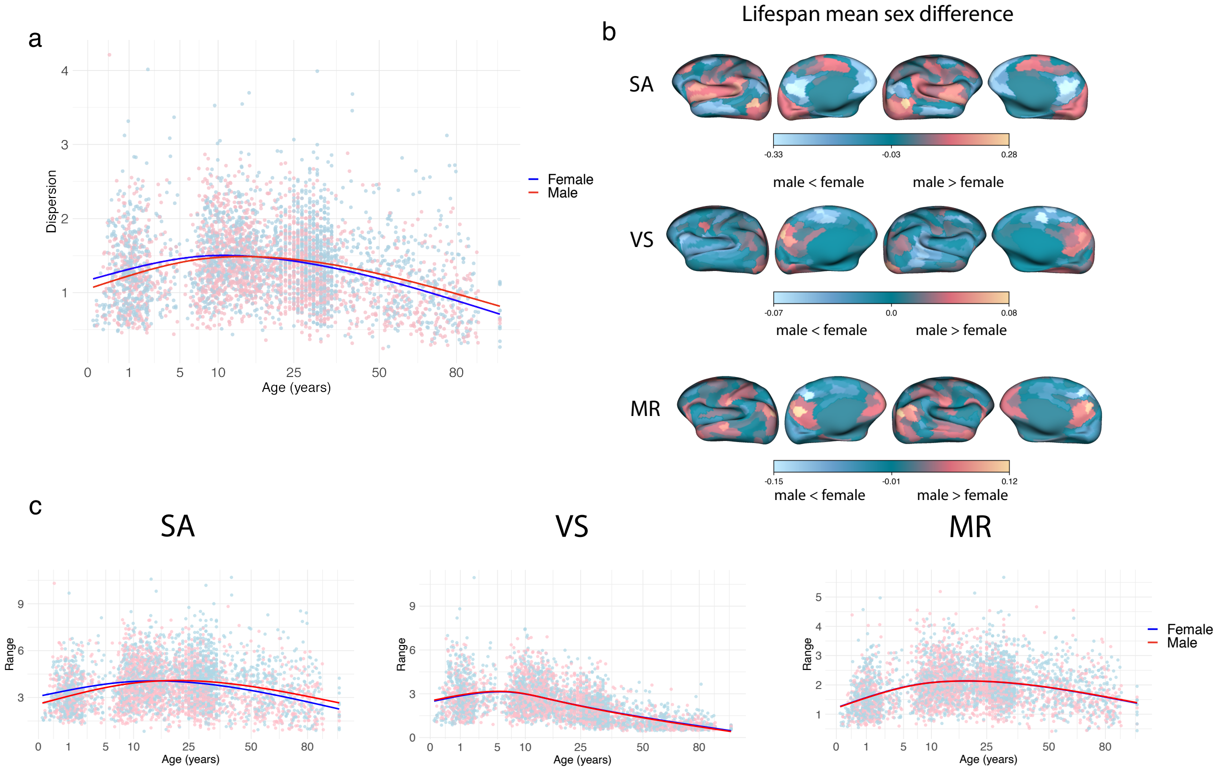

Supplementary Fig. 1 | **a**, GAMM fits of gradient dispersion for males and females. **b**, Surface maps of lifespan mean sex difference between parcellated gradient values. **c**, GAMM fits of SA, VS, and MR gradient ranges (from left to right) plotted against age for males and females.

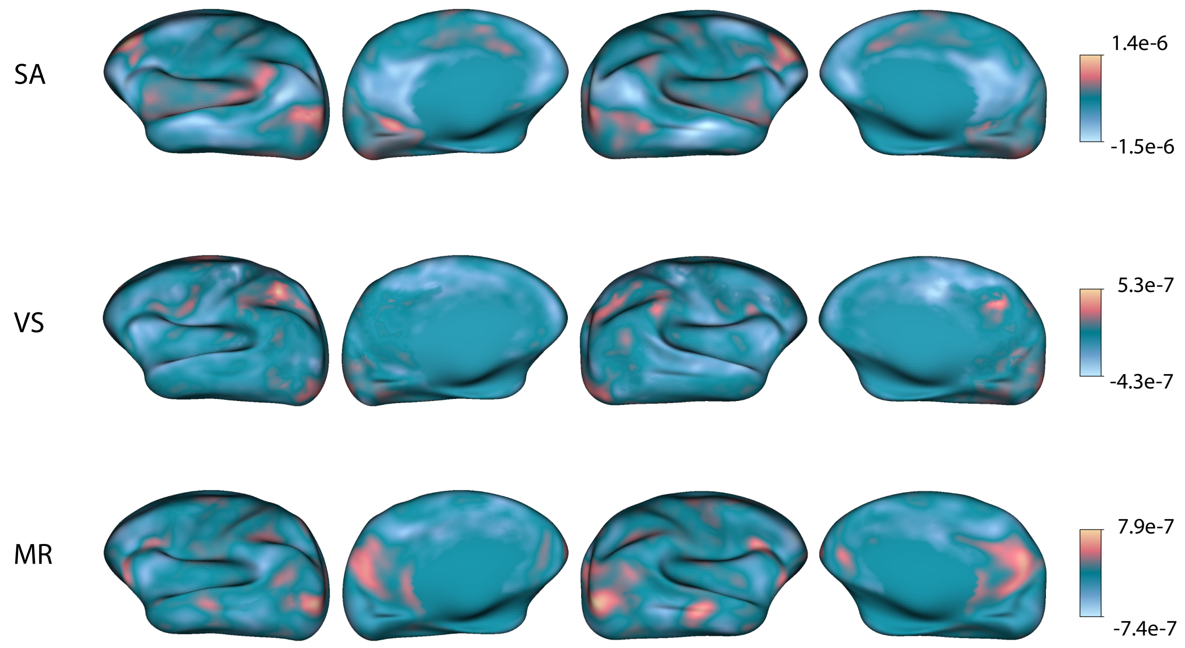

Supplementary Fig. 2 | Surface plots showing the effects of the total brain volume on vertex-wise gradient values across age.

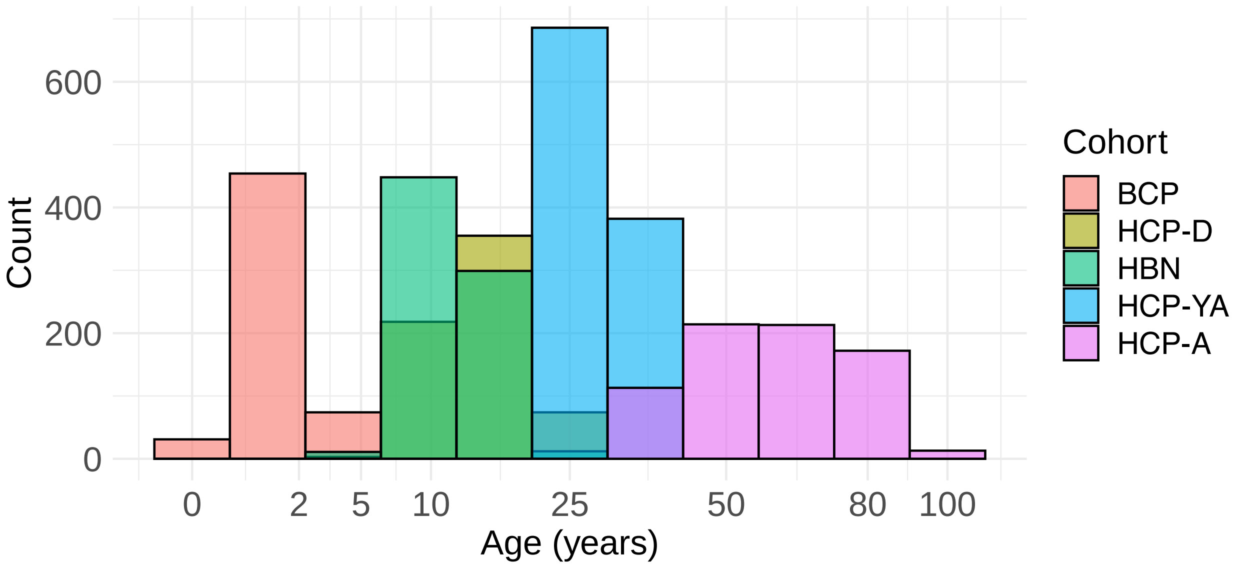

Supplementary Fig. 3 | Histogram of subject ages across all cohorts.

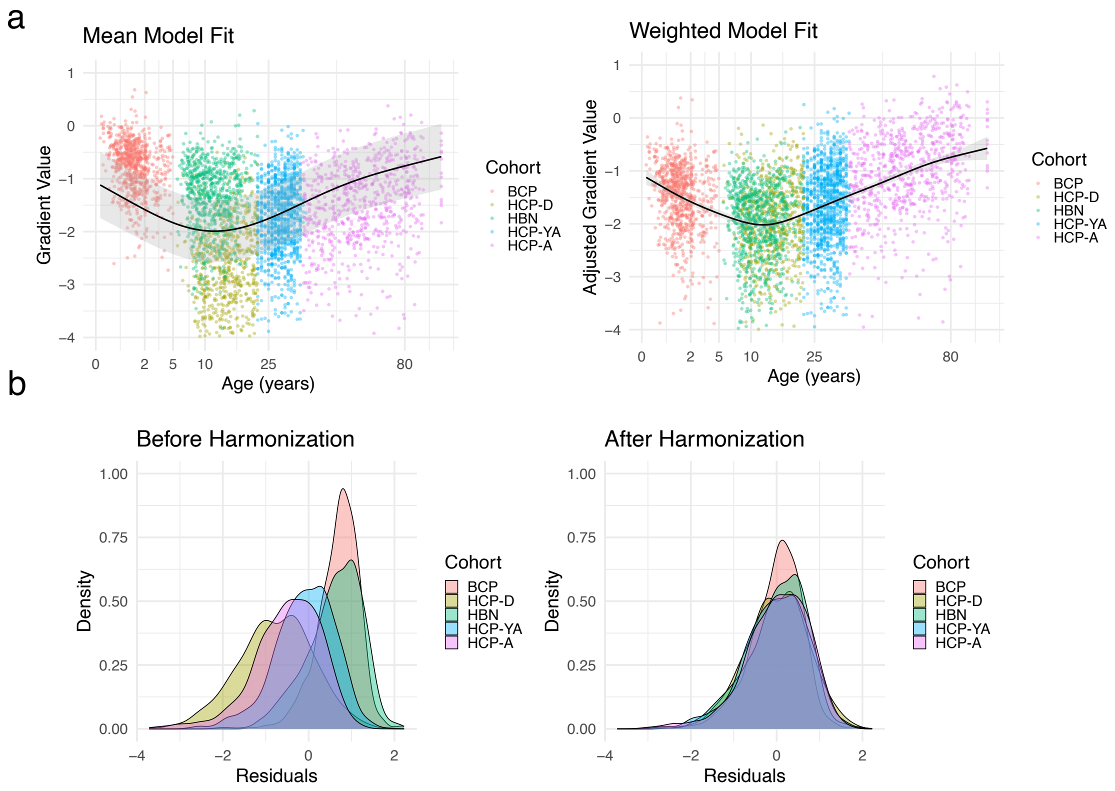

Supplementary Fig. 4 | Diagnostic plots for the harmonization procedure for an example vertex with a strong fit. **a**, Gradient values before harmonization for an example vertex with the initial mean model fit (left) and variance- and mean-adjusted gradient values with the final weighted model fit (right). **b**, Residual distributions by cohort before (left) and after (right) the harmonization procedure.

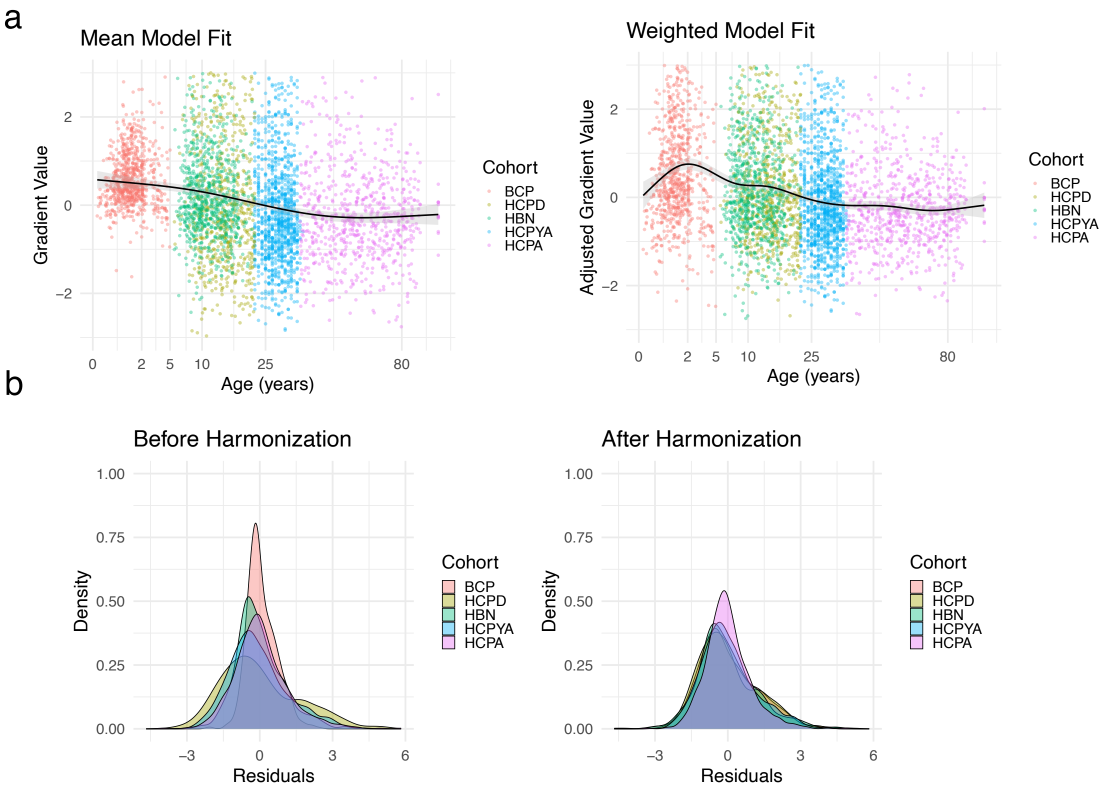

Supplementary Fig. 5 | Diagnostic plots for the harmonization procedure for an example vertex with a weaker GAMM fit. **a**, Gradient values before harmonization for an example vertex with the initial mean model fit (left) and variance- and mean-adjusted gradient values with the final weighted model fit (right). **b**, Residual distributions by cohort before (left) and after (right) the harmonization procedure.

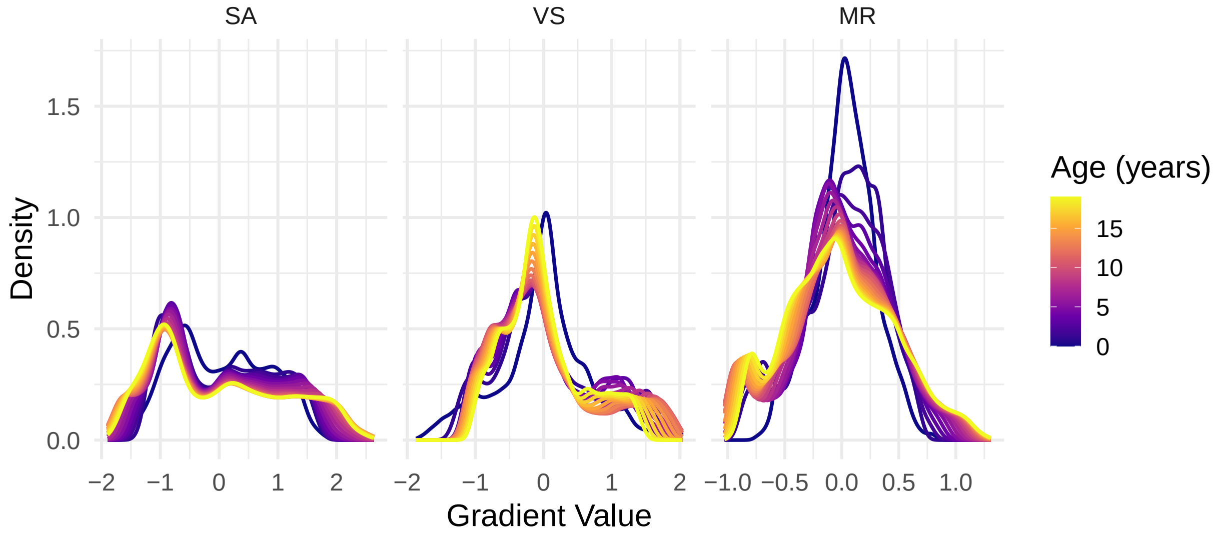

Supplementary Fig. 6 | Density plots for gradient value for the first 20 years of life plotted on the same axis and color-mapped to age for SA, VS, and MR gradients.

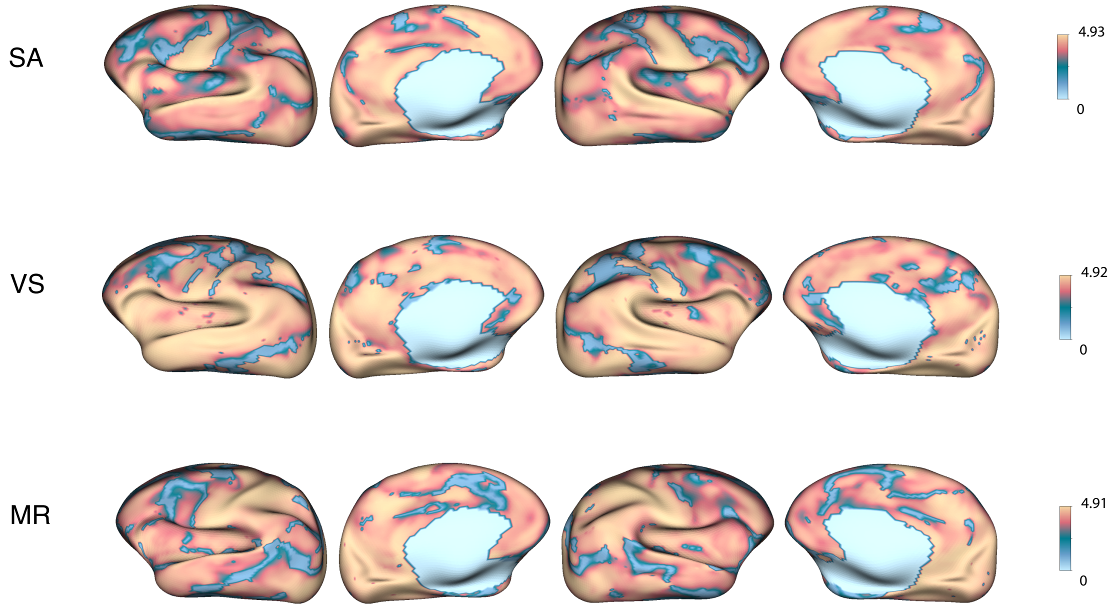

Supplementary Fig. 7 | Vertex-wise effective degrees of freedom values for GAMM fits of the SA, VS, and MR gradients.

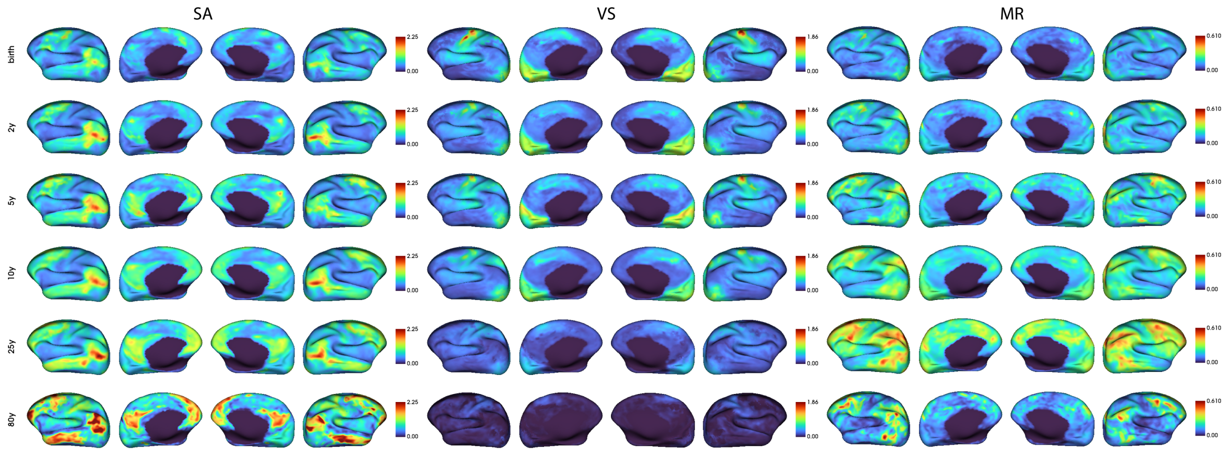

Supplementary Fig. 8 | Vertex-wise population sample variance of the aligned SA, VS, and MR gradients.

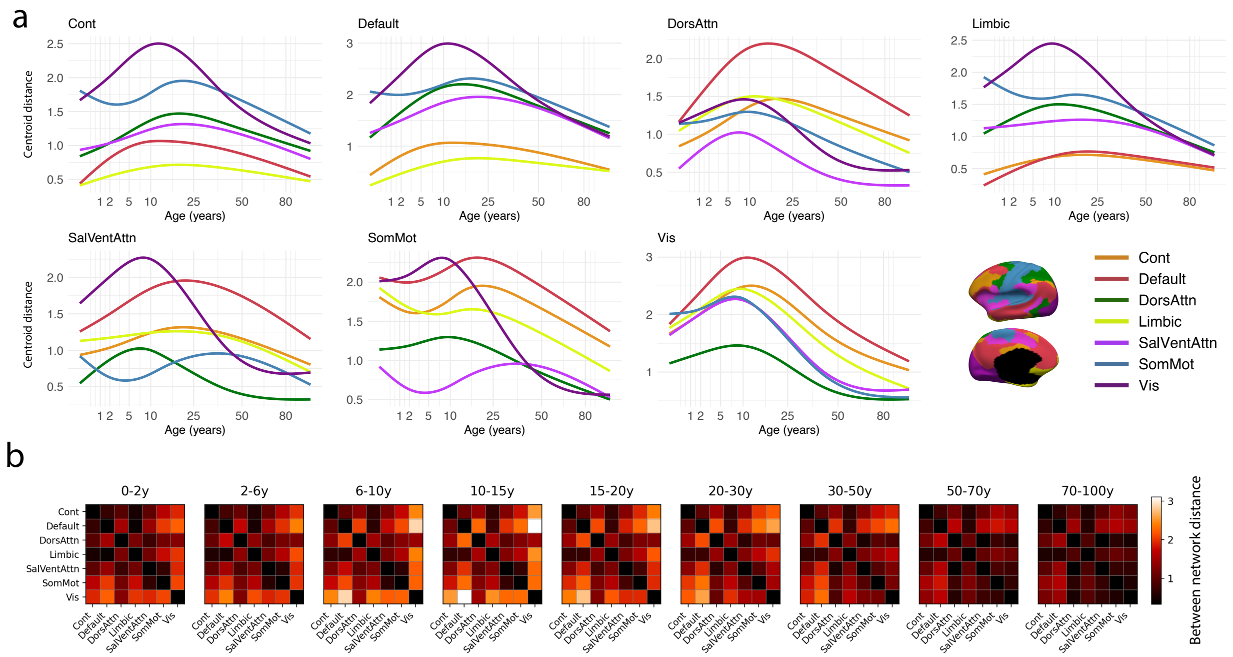

Supplementary Fig. 9 | GAMM fits of distance between resting-state network embedding centroids. Larger distances correspond to higher levels of functional differentiation and segregation. Small distances indicate functional similarity and integration. **a**, Each plot displays the lifespan GAMM fit of the Euclidean distance between network centroids in the SA–VS–MR gradient embedding space between the title network and each other network. **b**, Matrix plots of average network-network centroid distance in selected temporal windows, with lighter values denoting larger distance.

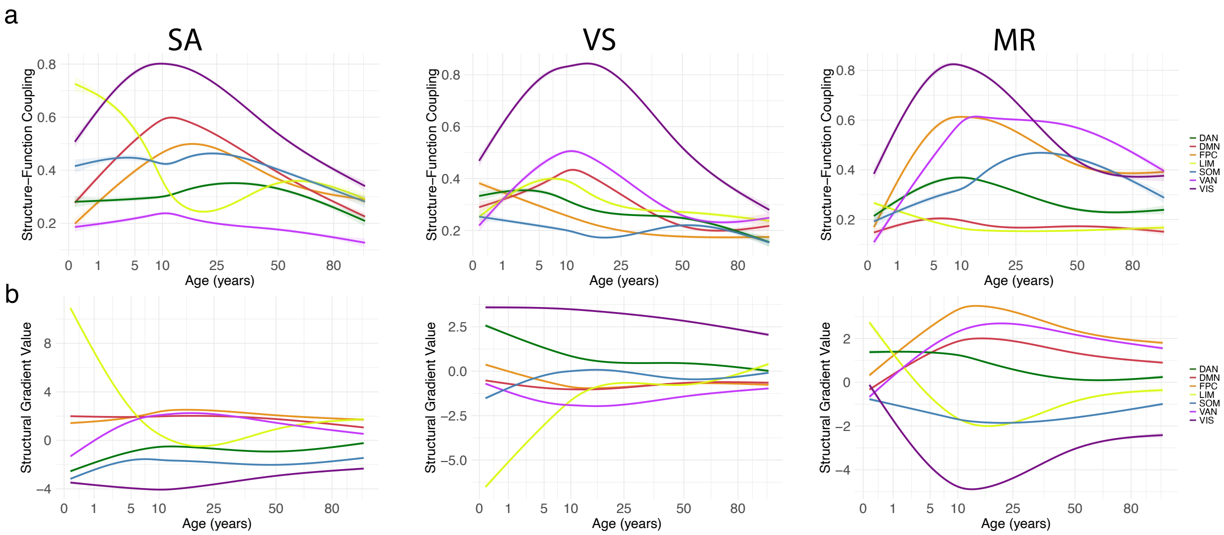

Supplementary Fig. 10 | Network-specific **a**, structure-function gradient coupling and **b**, structural gradient values based on the Schaefer 7-network parcellation.

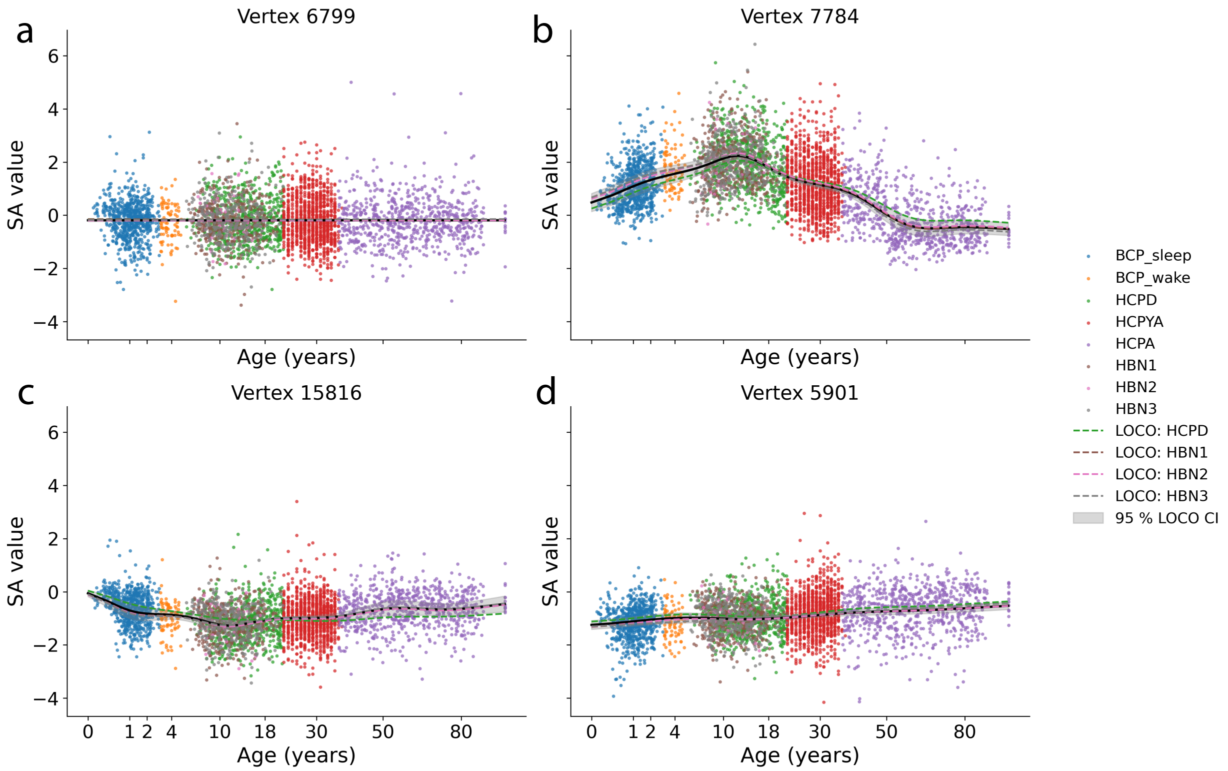

Supplementary Fig. 11 | Leave-one-cohort-out (LOCO) stability at four representative cortical vertices. Each panel shows the lifespan trajectory of the SA gradient at a single vertex after aligning gradients into the canonical space.ă Vertices were chosen to span the empirical range of signal amplitudes: **a**, the lowest-range vertex with a discernible signal, **b**, the highest-range vertex, and **c**,**d**, two vertices drawn at random from the inter-quartile amplitude band.

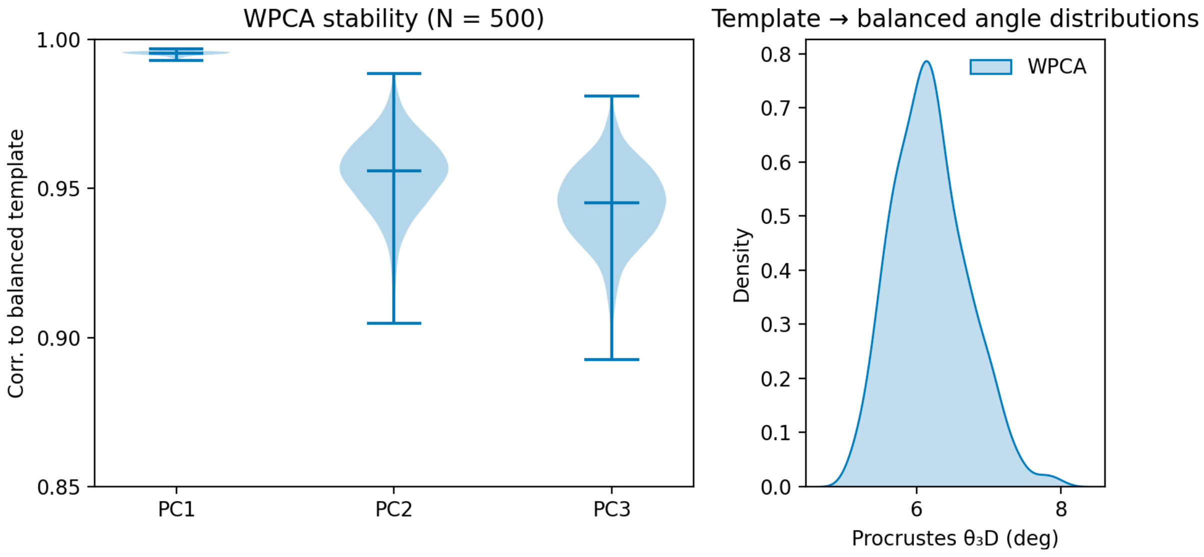

Supplementary Fig. 12 | Bootstrap validation of the WPCA gradient template. Left: Violin plots of per-axis correlations (PC1–PC3) between each balanced PCA template (50 subjects per bin, 10 α-transformed age bins, B = 500 bootstraps) and the canonical weighted PCA template. Right: Kernel density estimate of 3-D Procrustes subspace angles (θ) between the canonical WPCA template and each balanced-bootstrap template (mean $\theta = 6.18°$, SD = 0.52°).

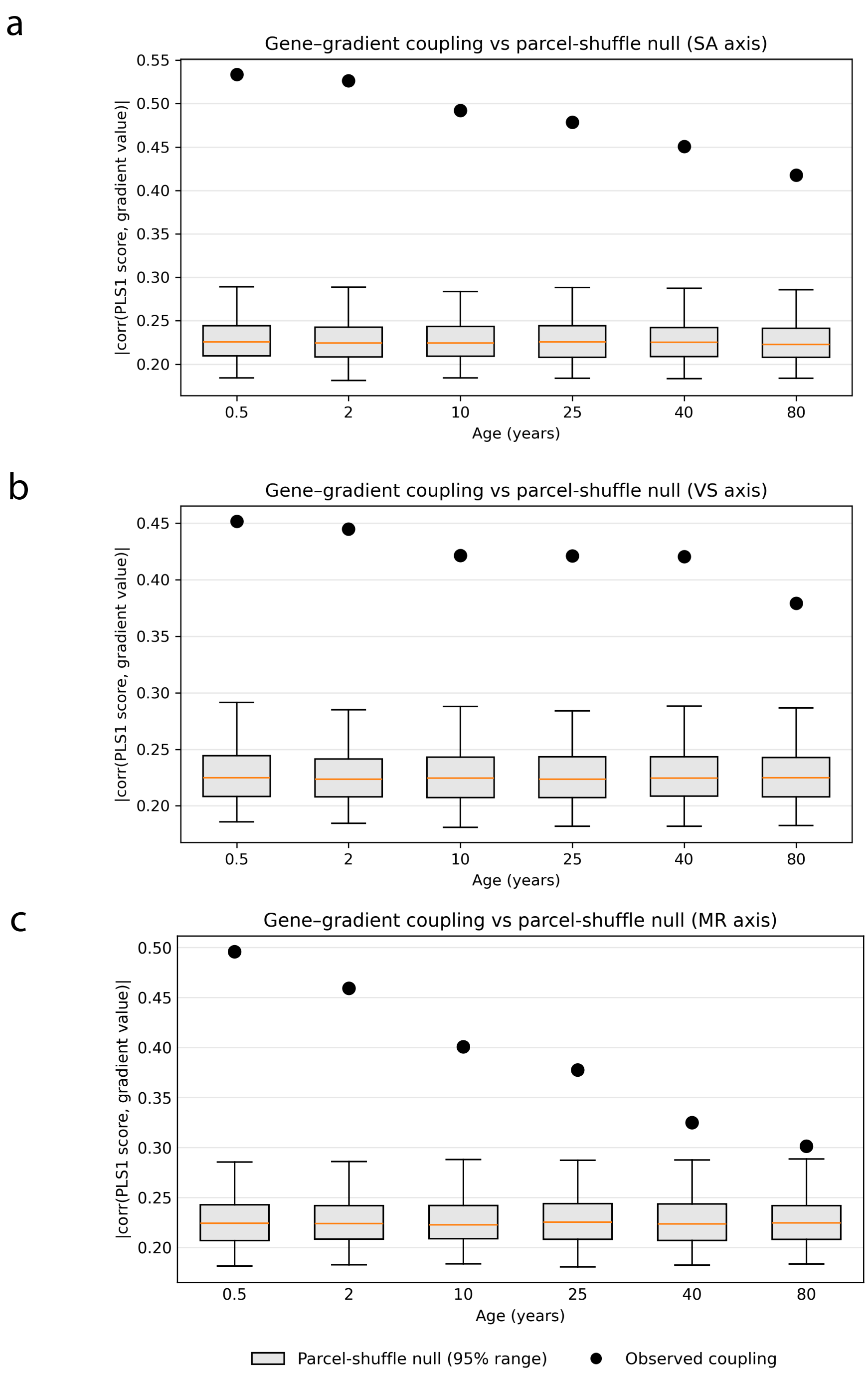

Supplementary Fig. 13 | Gene–gradient coupling compared to parcel-shuffle null models for each functional axis. a–c, For each age and axis, we quantified gene–gradient coupling as the absolute Pearson correlation between parcel-wise PLS1 scores and mean gradient values. Gray boxplots show the distribution of coupling values obtained under a parcel-shuffle null model, in which gradient values were randomly permuted across parcels (2,000 permutations per age), and whiskers denote the 2.5–97.5th percentiles. Black points show the observed coupling for the (a) SA, (b) VS, and (c) MR axes. For all three gradients and all ages, the empirical coupling exceeds the upper tail of the null distribution, confirming that the PLS associations reflect spatially specific gene–gradient alignment rather than parcellation idiosyncrasies.

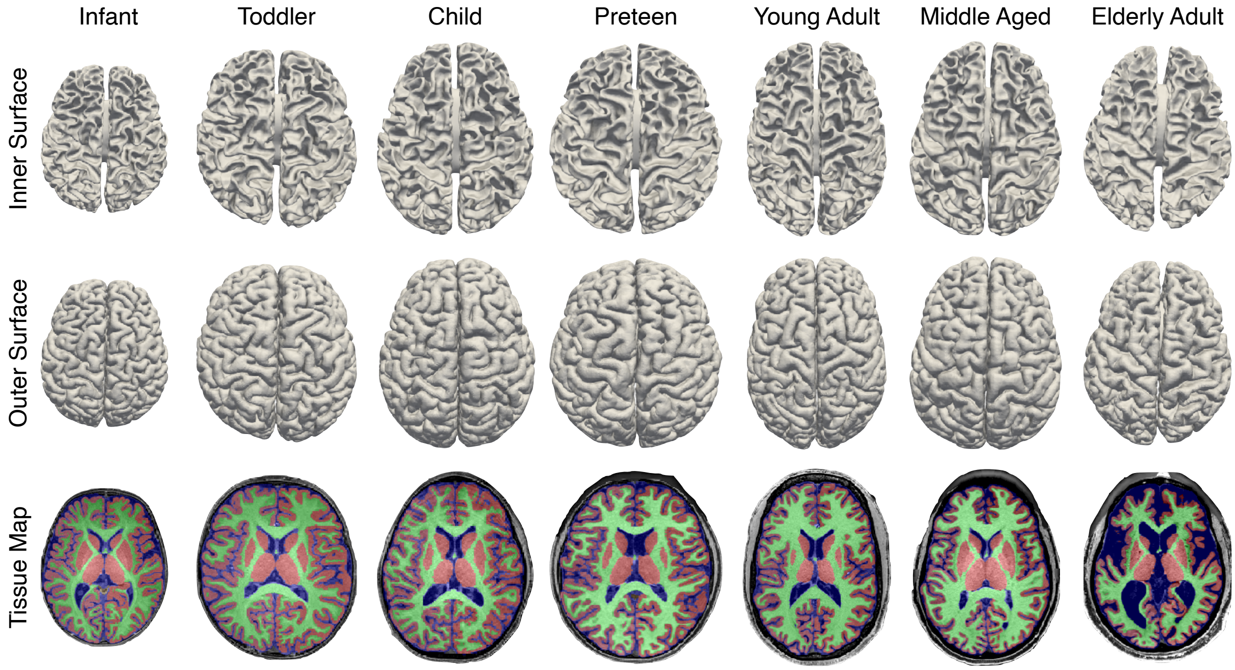

Supplementary Fig. 14 | Example tissue segmentation maps and cortical surfaces across the human lifespan.

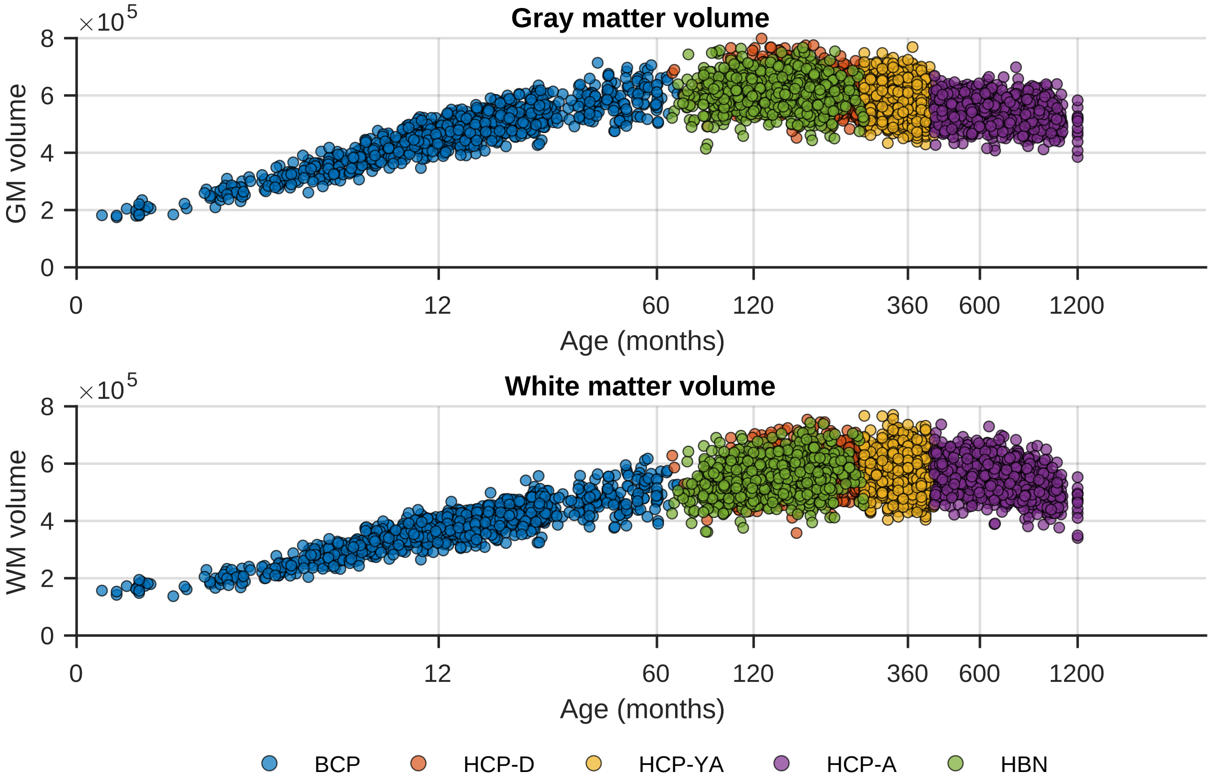

Supplementary Fig. 15 | Volumetric trajectories (in mm^3) of white matter and gray matter across the human lifespan.

Supplementary Tables

Supplementary Tab. 1 | Associations between Mullen Scales of Early Learning and gradient metrics in the BCP cohort (*n* = 239 subjects, 453 samples). The relationship between each Mullen score and each gradient metric was examined using linear mixed-effects models, controlling for age and including a random intercept for each participant. Each cell displays the estimated coefficient (β) for the gradient metric, along with the corresponding p-value. Entries with FDR-significant effects ($p_{FDR} < .05$) are bolded.

Metric	Gross	Fine	Visual	RecLang	ExpLang	Composite
Dispersion	0.01/0.93	-0.00/0.93	0.01/0.93	-0.01/0.93	-0.00/0.94	0.00/0.94
SA range	-0.01/0.93	-0.01/0.93	0.01/0.93	-0.01/0.93	0.00/0.98	0.00/0.93
VS range	0.01/0.91	-0.03/0.34	-0.01/0.93	-0.02/0.7	-0.00/0.94	-0.01/0.82
MR range	0.00/0.94	0.02/0.43	0.01/0.93	0.00/0.97	-0.00/0.93	0.01/0.77
SA cossim	-0.01/0.93	-0.00/0.93	0.02/0.67	0.02/0.7	0.03/0.34	0.02/0.54
VS cossim	0.01/0.93	0.02/0.48	0.03/0.34	0.02/0.43	0.01/0.77	0.02/0.23
MR cossim	-0.00/0.94	0.02/0.7	**0.05/0.021**	**0.05/0.021**	0.04/0.13	**0.04/0.021**
Eval1	0.01/0.93	0.01/0.84	0.01/0.81	-0.00/0.93	-0.01/0.93	0.01/0.93
Eval2	0.02/0.62	0.03/0.25	0.02/0.62	0.00/0.93	0.00/0.93	0.02/0.43
Eval3	0.04/0.24	**0.05/0.021**	0.03/0.34	0.02/0.7	0.00/0.93	0.03/0.17

Supplementary Tab. 2 | Associations between NIH Toolbox scores and gradient metrics in the HCP-YA cohort ($n = 1{,}066$). Each cell reports the standardised slope (β) and its BH-adjusted p-value (p_{FDR}). Entries with $p_{FDR} < .05$ are bolded.

Metric	Total	Fluid	Crystal	ListSort	Flanker	CardSort	ProcSpeed	ReadEng	PicSeq
Dispersion	**0.10/0.011**	**0.10/0.01**	**0.09/0.02**	0.02/0.57	0.06/0.081	0.04/0.28	**0.07/0.044**	0.07/0.08	**0.12/0.0016**
SA range	**0.10/0.011**	**0.10/0.009**	0.06/0.081	0.04/0.30	**0.08/0.038**	0.05/0.19	**0.08/0.032**	0.07/0.068	**0.11/0.0019**
VS range	-0.01/0.88	0.01/0.72	**0.08/0.022**	-0.05/0.18	0.02/0.62	-0.03/0.37	-0.02/0.54	-0.03/0.38	0.03/0.38
MR range	0.06/0.083	0.05/0.12	0.06/0.082	0.02/0.49	0.04/0.29	0.00/0.88	0.04/0.23	0.05/0.13	0.07/0.064
SA cossim	**0.19/1.8e-07**	**0.17/1.3e-06**	**0.12/0.0013**	**0.09/0.02**	**0.09/0.011**	**0.08/0.022**	**0.15/1.6e-05**	**0.15/3.5e-05**	**0.18/4.8e-07**
VS cossim	0.05/0.16	**0.08/0.023**	0.06/0.083	0.06/0.11	0.05/0.18	0.03/0.48	-0.01/0.80	-0.01/0.83	**0.08/0.032**
MR cossim	**0.10/0.011**	**0.11/0.0021**	**0.08/0.039**	0.06/0.082	0.06/0.11	0.05/0.16	0.05/0.16	0.06/0.12	**0.14/5e-05**
Eval1	**0.08/0.022**	**0.09/0.021**	0.06/0.082	0.02/0.54	0.06/0.081	0.04/0.23	**0.07/0.045**	0.06/0.083	**0.09/0.011**
Eval2	0.06/0.087	0.06/0.11	**0.09/0.013**	0.00/0.95	0.04/0.27	-0.01/0.70	0.04/0.23	0.04/0.22	**0.08/0.022**
Eval3	**0.08/0.033**	0.06/0.082	**0.10/0.0091**	0.01/0.86	0.05/0.15	-0.02/0.51	0.05/0.16	0.06/0.083	**0.09/0.020**

Supplementary Tab. 3 | Cohort-wise motion parameters.

**Cohort**	**Number of Subjects**	**Number of Scans**	**Median Mean FD** (mm)
BCP	343	1,932	0.2684
HCP-D	650	2,505	0.1371
HBN	770	1,533	0.1804
HCP-YA	1,068	4,121	0.1435
HCP-A	725	2,885	0.1667
