# Supplementary material for: Functional hierarchy of the human neocortex across the lifespan
Source: Nature. 2026 Mar 25;652(8111):955–64. doi: 10.1038/s41586-026-10219-x (PMC13102691; doi:10.1038/s41586-026-10219-x)
Supplement: Supplementary file 2 — Reporting Summary [file 41586_2026_10219_MOESM2_ESM.pdf]

nature portfolio

Corresponding author(s): Hoyt Patrick Taylor IV, Pew-Thian Yap

Last updated by author(s): Jan 21, 2026

Reporting Summary

Nature Portfolio wishes to improve the reproducibility of the work that we publish. This form provides structure for consistency and transparency in reporting. For further information on Nature Portfolio policies, see our Editorial Policies and the Editorial Policy Checklist.

Statistics

For all statistical analyses, confirm that the following items are present in the figure legend, table legend, main text, or Methods section.

n/a	Confirmed	
☐	☒	The exact sample size (*n*) for each experimental group/condition, given as a discrete number and unit of measurement
☐	☒	A statement on whether measurements were taken from distinct samples or whether the same sample was measured repeatedly
☐	☒	The statistical test(s) used AND whether they are one- or two-sided<br>*Only common tests should be described solely by name; describe more complex techniques in the Methods section.*
☐	☒	A description of all covariates tested
☐	☒	A description of any assumptions or corrections, such as tests of normality and adjustment for multiple comparisons
☐	☒	A full description of the statistical parameters including central tendency (e.g. means) or other basic estimates (e.g. regression coefficient) AND variation (e.g. standard deviation) or associated estimates of uncertainty (e.g. confidence intervals)
☐	☒	For null hypothesis testing, the test statistic (e.g. *F*, *t*, *r*) with confidence intervals, effect sizes, degrees of freedom and *P* value noted<br>*Give P values as exact values whenever suitable.*
☒	☐	For Bayesian analysis, information on the choice of priors and Markov chain Monte Carlo settings
☐	☒	For hierarchical and complex designs, identification of the appropriate level for tests and full reporting of outcomes
☐	☒	Estimates of effect sizes (e.g. Cohen's *d*, Pearson's *r*), indicating how they were calculated

Our web collection on statistics for biologists contains articles on many of the points above.

Software and code

Policy information about availability of computer code

Data collection	No software was used
Data analysis	Python libraries: Numpy 1.26.4, Scipy 1.13.1, Scikit-Learn 1.4.0, Nibabel 5.2.0, Brainspace 0.1.10, WPCA 0.1, Abagen 0.1.3, NiMare 0.5.5. fMRI + dMRI preprocessing: FSL 6.0, Connectome Workbench. SMSI microstructure fitting: Matlab 2022b R libraries: MGCV 1.8.39, dplyr 1.1.3, tidyr 1.3.0, ggplot2 3.4.4, readr 2.1.4, tibble 3.2.1, purrr 1.0.2. In-house surface atlas from Ahmad et al. 2022. Gene Ontology: GOrilla

For manuscripts utilizing custom algorithms or software that are central to the research but not yet described in published literature, software must be made available to editors and reviewers. We strongly encourage code deposition in a community repository (e.g. GitHub). See the Nature Portfolio guidelines for submitting code & software for further information.

Data

Policy information about availability of data

All manuscripts must include a data availability statement. This statement should provide the following information, where applicable:

- Accession codes, unique identifiers, or web links for publicly available datasets
- A description of any restrictions on data availability
- For clinical datasets or third party data, please ensure that the statement adheres to our policy

The Lifespan HCP fMRI data is publicly available via the National Institute of Mental Health data archive (NDA, http://nda.nih.gov). All the data are deposited under the Connectome Coordination Facility repository with the following Collection IDs (BCP: #2848; HCP-D: #2846; HCP-YA: #2825; HCP-A: #2847). The HBN MRI data

are available from https://healthybrainnetwork.org/. In addition, meta-analytic decoding resources were obtained from the Neurosynth database (version 7; abstract-based term annotations) downloaded via the NiMARE Python package (https://nimare.readthedocs.io/en/0.0.1/auto_examples/01_datasets/download_neurosynth.html) and converted to a NiMARE dataset. Allen Human Brain Atlas (AHBA) normalized microarray datasets (six donor brains) were downloaded from the Allen Institute portal and processed with abagen (https://abagen.readthedocs.io/en/stable/user_guide/download.html) to generate parcellated regional expression matrices.

Research involving human participants, their data, or biological material

Policy information about studies with human participants or human data. See also policy information about sex, gender (identity/presentation), and sexual orientation and race, ethnicity and racism.

Reporting on sex and gender	Subjects in the Baby Connectome Project (BCP) ranged from 16 days to 6 years old, with 343 subjects 158 males, 176 females, 9 unreported). We used 650 subjects (301 males, 349 females) from the HCP-D, 770 subjects from the HBN (458 males, 275 females, 37 unreported), 1068 subjects (482 males, 586 females) from the HCP-YA, and 725 subjects (319 males, 406 females) from the HCP-A. Across all four cohorts, our dataset included 3,556 subjects.
Reporting on race, ethnicity, or other socially relevant groupings	We did not use race, ethnicity, or any other socially relevant groupings.
Population characteristics	BCP subjects age ranged from 16 days to 6 years old. Subjects from the HCP-D ranged from 5.58-21.92 years old. Subjects in the HBN ranged from 5.57-21.9 years. Subjects from the HCP-YA ranged from 22-37 years old. Subjects from the HCP-A ranged from 36-100 years old. The median age across all participants was 26 years with a standard deviation of 21.33 years.
Recruitment	For BCP data, participants were recruited from existing registries at UNC and UMN based on state-wide birth records as well as from broader community resources (e.g., community centers and targeted day-care centers) to ensure the sample approximates the racial/ethnic and socio-economic diversity of the US census.
Ethics oversight	For BCP data, the study protocols were approved by the Institutional Review Board of the School of Medicine of the University of North Carolina at Chapel Hill (UNC-CH), NC, USA.

Note that full information on the approval of the study protocol must also be provided in the manuscript.

Field-specific reporting

Please select the one below that is the best fit for your research. If you are not sure, read the appropriate sections before making your selection.

☒ Life sciences ☐ Behavioural & social sciences ☐ Ecological, evolutionary & environmental sciences

For a reference copy of the document with all sections, see nature.com/documents/nr-reporting-summary-flat.pdf

Life sciences study design

All studies must disclose on these points even when the disclosure is negative.

Sample size	Sample size was chosen based on available data across our five imaging datasets. Our final sample size of 3972 is large and ages of participants are evenly distributed across the human lifespan.
Data exclusions	Datapoints for which cortical surface meshes with vertex correspondence across the lifespan could not be constructed were excluded from subsequent analyses. Subjects for which fMRI motion parameters exceeded mean Power's FD (absolute sum of motion parameters) exceeding 0.5mm—a common threshold in fMRI studies, were excluded. Finally, we used visual inspection and a clustering procedure to exclude subjects with non-biological or corrupted FC gradients.
Replication	We evaluated reproducibility of key lifespan-derived quantities (trajectory shape, peak age, and gradient template definition) using multiple stability analyses. Peak-age estimates from GAMM fits of gradient metrics were reproducible across 20,000 coefficient-draw simulations (parametric bootstrap), vertex-wise trajectories were robust in 4 leave-one-cohort-out refits with good held-out generalization, and the WPCA template used for alignment was stable across 500 age-balanced bootstrap replicates.
Randomization	Our study does not involve the division of our dataset into multiple groups. Instead, we are tracking how functional connectivity organization changes with respect to age. Thus, no randomization was required.
Blinding	This study is observational and analyzes previously acquired datasets spanning the lifespan. Participants were not assigned to experimental conditions or intervention groups; therefore, randomization was not applicable and was not performed.

Reporting for specific materials, systems and methods

We require information from authors about some types of materials, experimental systems and methods used in many studies. Here, indicate whether each material, system or method listed is relevant to your study. If you are not sure if a list item applies to your research, read the appropriate section before selecting a response.

Materials & experimental systems

n/a	Involved in the study
☒	☐ Antibodies
☒	☐ Eukaryotic cell lines
☒	☐ Palaeontology and archaeology
☒	☐ Animals and other organisms
☒	☐ Clinical data
☒	☐ Dual use research of concern
☒	☐ Plants

Methods

n/a	Involved in the study
☒	☐ ChIP-seq
☒	☐ Flow cytometry
☐	☒ MRI-based neuroimaging

Plants

Seed stocks: N/A

Novel plant genotypes: N/A

Authentication: N/A

Magnetic resonance imaging

Experimental design

Design type: resting-state

Design specifications: For each subject/timepoint (some subjects in the BCP were scanned at multiple ages in staggered-cohort longitudinal study design), 4 resting-state sessions were carried out. For the BCP, each session contained 478 TRs, with TR = 800ms . For the HCP-D and HCP-A, each session contained 420 TRs, with TR = 800ms . For the HCP-YA, each session contained 1200 TRs, with TR = 720ms. For the HBN, each session contained 375 TRs, with TR = 800ms.

Behavioral performance measures: Mullen Scales of Early Learning were used for BCP. NIH toolbox cognition battery was used for HCPYA, HCPD, and HCPA.

Acquisition

Imaging type(s): functional, structural, and diffusion.

Field strength: 3T

Sequence & imaging parameters:

BCP sMRI data: MPRAGE, SPACE, FOV: 256mm x 256mm, slice thickness: 0.8mm.
BCP rfMRI: 2D multiband gradient-recalled echo-planar imaging (flip angle 52 degrees), FOV: 208mm x 208mm slice thickness: 2mm.
BCP dMRI: Single-shot EPI, FOV 210mm x 210 mm, slice thickness: 1.5mm, 6 shells, 144 gradient directions

HCPD + HCPA sMRI: MPRAGE, SPACE, FOV: 256mm x 240mm x 160mm, slice thickness: 0.8mm.
HCPD + HCPA fMRI: 2D multiband gradient-recalled echo-planar imaging (flip angle 52 degrees), slice thickness: 2mm, with 72 oblique-axial slices.
HCPD + HCPA dMRI: slice thickness: 1.5mm, 2 shells, 180 gradient directions

HCPYA sMRI:MPRAGE, SPACE, FOV 224mm x 224mm, slice thickness: 0.7mm.
HCPYA fMRI: Gradient-echo EPI, FOV: 208mm x 180mm, slice thickness: 2mm
HCPYA dMRI: Spin-echo EPI, FOV 210mm x 180mm, slice thickness 1.25mm, 3 shells, 270 gradient directions

HBN sMRI: MPRAGE, SPACE, FOV 224mm x 224mm, slice thickness: 0.8mm or 1mm depending on scanning sites.
HBN fMRI: Gradient-echo EPI, FOV: 208mm x 208mm, slice thickness: 2.4mm
HBN dMRI: Spin-echo EPI, FOV 208mm x 208mm, slice thickness 1.8mm, 2 shells, 128 gradient directions

Area of acquisition: whole-brain scans were acquired.

Diffusion MRI: ☒ Used ☐ Not used

Parameters	BCP: 144 directions, 6 shells with b = 500, 1000, 1500, 2000, 2500, 3000, AP and PA encoding directions<br>HCPD + HCPA: 180 directions, 2 shells with b = 1500, 3000, AP and PA encoding directions<br>HCPYA: 270 directions, 3 shells with b = 1000, 2000, 3000, LR and RL encoding directions<br>HBN: 128 directions, 2 shells with b = 1000, 2000, PA encoding direction with 1 AP B0

Preprocessing

Preprocessing software	Preprocessing involved an in-house pipeline consistent with the HCP minimal preprocessing pipeline suited for infant neuroimaging data. Softwares used included FSL and Freesurfer.
Normalization	Rigid transform
Normalization template	In-house brain template
Noise and artifact removal	Bias field correction for sMRI data. ICA-AROMA was used for rsfMRI nuisance regression.
Volume censoring	Not applicable.

Statistical modeling & inference

Model type and settings	Statistical analyses used generalized additive models/mixed models (GAM/GAMM) in R (mgcv; bam with fast REML for large models) with penalized regression spline smooths; smoothing parameters were estimated by (fast) REML. Lifespan models used a monotonic age reparameterization (square root of age). Vertex-wise lifespan models included random intercepts for cohort and subject ID (to account for between-cohort differences and repeated measures in BCP), sample-density weights to balance age bins (and cohort size), and a two-step heteroscedasticity correction (GAMM on squared residuals + inverse variance weighting in the final fit; basis dimension tuned with low-k harmonization and higher-k final fits). Additional models included ordinary least squares regression for cross-sectional HCP-YA cognition, linear mixed-effects models for repeated-measures BCP cognition, and partial least squares regression (PLS1; scikit-learn) with permutation testing for transcriptomic analyses.
Effect(s) tested	Primary effects tested were age-dependent changes in gradient values/metrics (smooth age terms) and departures from linear age effects (per-vertex LM vs GAM partial F-tests with FDR correction across vertices). Sex differences were assessed via sex-specific deviations from the population trajectory using sex-by-age smooths. Potential confounding by total brain volume was evaluated by refitting vertex-wise models including total brain volume as a covariate. Cognition analyses tested associations between gradient metrics and cognitive scores while adjusting for age (β_grad in OLS/LMM; smooth main effect of z(gradient) in GAM), and tested age modulation of these associations via an age×gradient interaction (tensor interaction ti(Age, z(gradient)) in GAM). Multiple comparisons were controlled using Benjamini–Hochberg FDR within each analysis family (e.g., across vertices or across cognition×metric tests).

Specify type of analysis: ☐ Whole brain ☐ ROI-based ☒ Both

Anatomical location(s)	We used cortical surface meshes and previously studied network and whole brain parcellations from Yeo et al. 2011 and Schaefer et al. 2018 , respectively.

Statistic type for inference<br>(See Eklund et al. 2016)	We used frequentist inference. Lifespan trajectories were modeled with GAMMs/GAMs (mgcv/gamm4; bam with fast REML) using penalized cubic regression splines (e.g., bs="cs") with smoothing parameters estimated by (fast) REML. Significance of smooth terms and tensor interactions was assessed using mgcv's approximate (Wald-type) tests (reported as F statistics for Gaussian models). Nonlinearity at each vertex was assessed via partial F-tests comparing nested models (linear vs smooth GAM). For cross-sectional HCP–YA cognition, we used ordinary least squares (lm) and tested regression coefficients using two-sided t-tests. For BCP cognition with repeated measures, we used linear mixed-effects models (lme4::lmer) with Satterthwaite degrees of freedom (lmerTest) for fixed-effect t-tests. Transcriptomic PLS models were evaluated using permutation testing of the in-sample correlation. All tests were two-sided unless stated otherwise.
Correction	Multiple comparisons were controlled using the Benjamini–Hochberg false discovery rate (FDR) procedure (q=0.05) within each analysis family. Specifically: (i) vertex-wise nonlinearity p-values (linear vs smooth GAM) were FDR-corrected across cortical vertices (separately for each gradient axis); (ii) HCP–YA cognition regressions were FDR-corrected across 9 cognitive scores × 10 gradient metrics (90 tests); (iii) BCP Mullen mixed models were FDR-corrected across 6 outcomes × 10 gradient metrics (60 tests); and (iv) transcriptomic permutation p-values were FDR-corrected across ages (within each gradient axis). No cluster-based correction was used.

Models & analysis

n/a	Involved in the study
☐	☒ Functional and/or effective connectivity
☐	☒ Graph analysis
☒	☐ Multivariate modeling or predictive analysis

Functional and/or effective connectivity	We used Pearson's correlation coefficient to measure functional connectivity.
Graph analysis	We used weighted functional connectivity graphs (pairwise Pearson correlation coefficient) and computed their diffusion map embeddings (referred to in manuscript as functional connectivity gradients). We

performed auxiliary analysis on the functional connectivity graphs that included computing functional connectivity degree (row-wise sum of thresholded FC matrices).
